# Conserved Pathways, Divergent Outcomes: A Cross-Species Genomic Perspective on the Cancer–Neurodegeneration Paradox

**DOI:** 10.3390/ijms27072989

**Published:** 2026-03-25

**Authors:** Bhargavi Rajarathinam, Durga Nandan, Parvathy Venugopal, Amritha M. Nair, Subin John, Bipin G. Nair, Rajaguru Aradhya

**Affiliations:** School of Biotechnology, Amrita Vishwa Vidyapeetham, Kollam 690525, Kerala, India; bagi.eutopia@gmail.com (B.R.); bhilae@gmail.com (D.N.); parvathyv@am.amrita.edu (P.V.); amrithamnair2002@gmail.com (A.M.N.); subinjohn02@gmail.com (S.J.)

**Keywords:** evolutionary paradox, cross-species genomics, Peto’s paradox, antagonistic pleiotropy, tumor suppressor genes, comparative oncology, neuroprotection

## Abstract

Neurodegeneration and cancer are fundamentally distinct disorders: one signifies gradual neuronal loss while the latter signifies uncontrolled cell growth and survival. However, emerging evidence explores an inverse association between these conditions, suggesting that they do not arise from independent biological processes. Understanding the context-dependent behaviour of major pathways (for example, p53, PI3K/AKT/mTOR, Wnt, and immune–stress signaling) remains pivotal in elucidating the relationship between these two diseases. Pathways promoting early-life fitness, tissue repair, and tumor suppression in dividing cells can become detrimental later in life for post-mitotic neurons. Cross-species genomics studies reveal how evolution has repeatedly adapted these shared networks to balance cancer resistance with survival. Research on species exhibiting exceptional longevity and disease resistance, including naked mole rats and bowhead whales, shows that cancer resistance and longevity are not fixed traits but rather are controlled by precise regulatory mechanisms. In this review, we integrate insights from broad species genomics and multi-omic and single-cell studies to understand how evolutionarily conserved molecular crosstalks diverge at the interface of cancer and neurodegeneration.

## 1. Introduction

Cancer and neurodegeneration represent fundamentally opposing biological phenomena: uncontrolled cell proliferation versus progressive neuronal loss. Epidemiological research reveals a fascinating inverse correlation between these diseases [1]. Patients diagnosed with Alzheimer’s disease (AD) and Parkinson’s disease (PD) exhibit a reduced incidence of various cancers, and conversely, cancer survivors often show a decreased incidence of neurodegenerative disorders [2,3,4]. This complex interplay suggests that susceptibility to one disease may confer defense against the other, although exceptions exist, such as the elevated melanoma risk observed in PD patients [5]. Cancer therapies further complicate this relationship; for instance, chemotherapy in breast cancer survivors has been linked to both cognitive impairment and, yet, paradoxically, reduced subsequent AD risk, highlighting the multifaceted nature of disease interactions influenced by diagnosis, therapy, and survival [6,7].

At the molecular level, cancer and neurodegenerative diseases (NDDs) share numerous dysregulated genes, proteins, and signaling pathways, often exhibiting inverse expression patterns. For example, the tumor suppressor p53 is typically upregulated in NDDs such as AD, PD, and Huntington’s disease (HD), yet downregulated or mutated in most cancers [8,9]. Transcriptomic meta-analyses have demonstrated significant overlaps where genes upregulated in neurodegeneration are downregulated in cancer, and vice versa [10]. Despite these opposing trends, both disease groups share common pathophysiological features including inflammation, metabolic dysregulation, oxidative stress, DNA damage, and aberrant cell cycle activation [1]. Key genes implicated in both disease categories include PINK1, α-synuclein, PTEN, DJ-1, PSEN1/2, CDK5, LRRK2, APP and MAPT, highlighting a shared molecular framework with divergent functional outcomes [11].

At the cellular level, neurodegenerative disorders are characterized by hallmark features such as impaired proteostasis, protein misfolding, mitochondrial dysfunction, and chronic neuroinflammation. Aggregates of β-amyloid and tau in AD, α-synuclein in PD, and TDP-43 and FUS inclusions in amyotrophic lateral sclerosis (ALS) exemplify pathological protein accumulation [12,13]. Central to both neurodegeneration and cancer suppression are mitochondrial stress responses and defective autophagy mechanisms, particularly involving PINK1, Parkin, and DJ-1 [14]. While impaired mitophagy in neurons leads to reactive oxygen species (ROS) accumulation and apoptosis, in cancer cells, enhanced mitophagy supports metabolic adaptation and survival [15,16]. Rare “degenerative cancers,” such as glioblastoma multiforme, illustrate the coexistence of tumor proliferation alongside local neuronal degeneration, further blurring the boundaries between these disease processes [17,18]. Molecular players like apolipoprotein E (ApoE) further demonstrate how pathways involved in neurodegeneration also modulate tumorigenesis [19].

From an evolutionary perspective, the inverse relationship between cancer and neurodegeneration can be understood through the lens of evolutionary trade-offs and antagonistic pleiotropy. Certain pathways such as mTOR/IGF-1 and DNA repair mechanisms act as “double-edged swords” where adaptations beneficial in early life become detrimental with aging. For example, mTOR signaling promotes tumor growth by regulating metabolism and proliferation but, when hyperactivated, impairs autophagy contributing to neurodegenerative pathology. Similarly, p53 maintains genomic integrity and suppresses cancer by orchestrating DNA repair and apoptosis; however, constitutive p53 activation accelerates aging and neuronal loss [8,20]. Mouse models with augmented p53 activity demonstrate enhanced tumor resistance but premature aging phenotypes, illustrating antagonistic pleiotropy where evolutionary benefits come at the cost of late-life decline [20].

This review adopts a cross-species genomics perspective to explore how conserved molecular pathways governing cancer resistance and neurodegenerative vulnerability have evolved across diverse taxa, including humans, elephants, naked mole-rats, whales, and birds. By integrating comparative genomic data with mechanistic insights, it highlights how identical molecular networks can be fine-tuned toward tumor suppression, neural preservation, or, alternatively, late-life degeneration. Natural models exhibiting exceptional longevity and disease resistance provide valuable translational insights for balancing cancer prevention and neuroprotection, advancing therapeutic strategies that modulate shared pathways to mitigate both disease burdens.

## 2. Evolutionary Trade-Offs in Cancer and Neurodegeneration

Disease risk often reflects evolutionary adaptations optimized for early-life survival and reproduction rather than lifelong cellular stability [21]. Key cellular pathways regulating growth, stress responses, immune defense, and genome maintenance have been shaped by natural selection to maximize reproductive fitness, but these adaptations carry delayed costs that contribute to age-related diseases [22].

Cancer and neurodegeneration illustrate this trade-off: in spite of their different outcomes—uncontrolled growth versus gradual cell death—both result from dysregulation of conserved molecular programs [23]. Enhanced proliferative capacity or tumor suppression early in life can predispose to oncogenesis or neuronal vulnerability with aging [24]. This antagonistic pleiotropy framework explains the inverse epidemiological relationship observed between these diseases [25,26].

The following sections explore how conserved cellular systems are differentially engaged across tissues, cell types, and life stages to influence disease susceptibility.

### 2.1. Conserved Growth and Survival Pathways

Conserved molecular circuits regulate growth, metabolism, genome surveillance, and stress responses. Their biological effect varies depending on cellular context (proliferative versus post-mitotic (PM)), temporal stage (development versus aging), and the surrounding metabolic and inflammatory milieu [8]. This framework links the molecular frameworks of cancer and neurodegeneration as manifestations of evolutionary trade-offs embedded within conserved cellular systems [26,27].

#### 2.1.1. mTOR/IGF-1 Signaling: Proliferation Versus Proteostasis

The mTOR/IGF-1 signaling pathway regulates anabolic growth and cellular survival and functions as a key determinant of cellular fate across tissues. The mammalian target of rapamycin (mTOR), a serine/threonine kinase, processes signals from nutrient availability, growth factors, and cellular energy conditions to manage metabolism, autophagy, immune responses, protein synthesis, survival, proliferation, and migration, thereby maintaining cellular equilibrium [28]. Increased activation of mTOR signaling is a frequent hallmark of numerous human malignancies—including breast, lung, pancreatic, liver, renal, and prostate cancers—where it drives tumor growth by enhancing growth factor receptor signaling, glycolysis, lipid metabolism, angiogenesis, and therapy resistance [29,30]. These proliferative and metabolic advantages likely provided strong selective benefits early in life by supporting tissue growth, wound repair, and immune competence.

**Figure 1:**
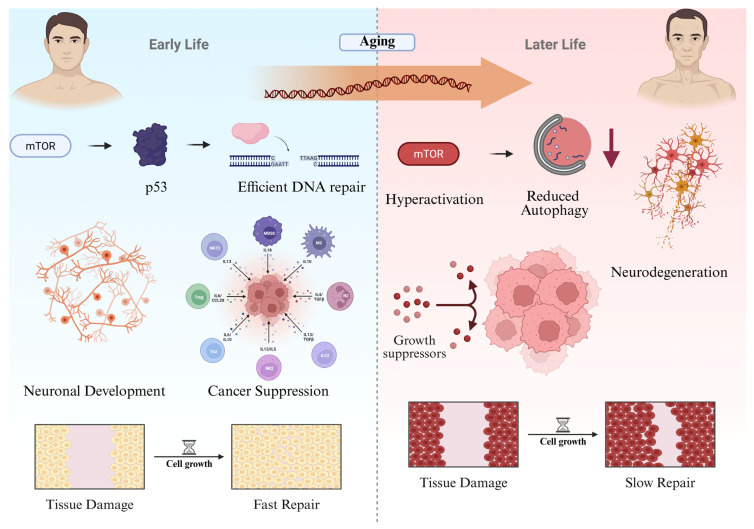
From Regeneration to Degeneration: Lifespan Trade-Offs in Conserved Growth and Stress Pathways.

Conversely, in the nervous system, chronic mTORC1 activation impairs autophagy, reducing clearance of damaged proteins and mitochondria. This leads to accumulation of aggregation-prone species like β-amyloid, tau, and α-synuclein, which drive NDDs such as AD and PD [31]. The inverse relationship between mTORC1 activity and autophagy is well established; pharmacological or genetic inhibition of mTORC1 restores autophagic flux and provides neuroprotection in various models [32]. This contrast illustrates how a pathway that promotes proliferation and survival in dividing tissues can disrupt homeostasis and accelerate degeneration when persistently active in post-mitotic neurons (Figure 1). Comparative studies across species further suggest that evolutionary modulation of mTOR signaling is a recurring strategy to balance cancer resistance with neural maintenance in long-lived mammals.

#### 2.1.2. p53-Mediated Genome Surveillance: Cancer Suppression Versus Neuronal Loss

As a central genome surveillance module, p53 maintains genomic integrity by sensing genotoxic stress and coordinating DNA repair, transient cell-cycle arrest, senescence, or apoptosis to prevent propagation of damaged cells in proliferative tissues [33,34]. Loss-of-function mutations in TP53 are among the most frequent variations in cancers, disabling tumor suppression and, in many cases, conferring gain-of-function activities that actively promote cell growth, survival, and metastasis [35]. Notably, mutant p53 proteins can suppress the expression of apoptotic receptors such as CD95 (Fas/APO-1), thereby exerting antiapoptotic effects that facilitate tumor progression [36].

From an evolutionary perspective, robust p53 activity confers substantial early-life advantages by maintaining genomic integrity, limiting malignant transformation, and supporting reproductive success [37]. However, sustained or constitutive p53 activation accelerates aging-related decline. Mouse models engineered to express augmented wild-type p53 activity (p53+/m) exhibit enhanced resistance to spontaneous tumors yet develop premature aging phenotypes, including reduced longevity, osteoporosis, organ atrophy, and reduced stress tolerance [37,38,39].

In PM neurons, which are energetically demanding and largely irreplaceable, chronic or excessive p53 activation promotes apoptosis, synaptic loss, and neurite degeneration rather than cell-cycle arrest [40,41]. Aging-associated accumulation of DNA damage, mitochondrial dysfunction, and oxidative stress sustains p53 signaling in vulnerable neuronal populations, converting a cancer-protective genome surveillance mechanism into a driver of neurodegeneration [42]. These findings underscore the importance of tightly regulated p53 activity across tissues and lifespan.

##### Integrative Perspective

mTOR and p53 illustrate how conserved growth and stress-response pathways generate divergent outcomes across tissues and lifespan. In dividing cells, sustained signaling may support malignant expansion; in PM neurons, prolonged activation may impair proteostasis and promote cell loss.

This view aligns with geroscience frameworks proposing that aging-associated diseases arise from dysregulation of interconnected systems governing growth signaling (mTOR/IGF-1), genome integrity (p53), mitochondrial homeostasis, protein quality control, and inflammation [43,44]. Therapeutic strategies therefore require tissue-specific and age-dependent modulation of these networks.

### 2.2. Antagonistic Pleiotropy Across Cell Types

**Figure 2:**
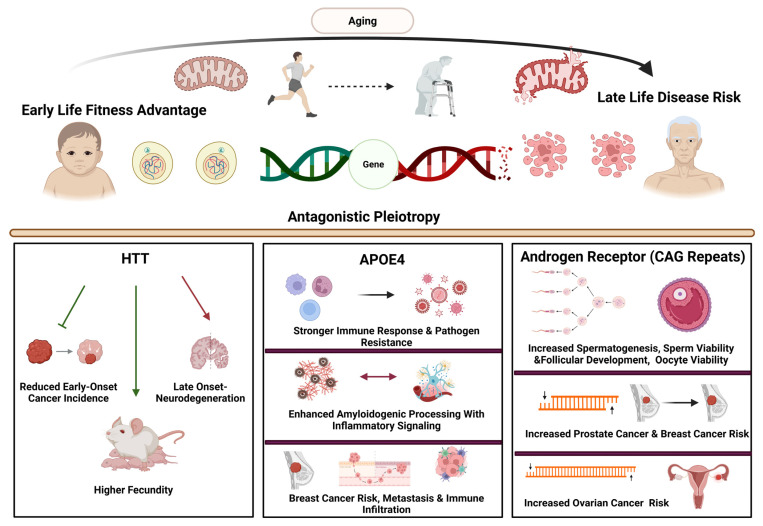
Schematic representation of antagonistic pleiotropy across species and cell types: Genetic variants promoting early-life proliferation and late-life neurodegeneration. Arrows indicate biological progression or causal relationships between processes, while bidirectional arrows represent reciprocal interactions, curved arrows indicate progression of aging across the lifespan.

Antagonistic pleiotropy provides an evolutionary framework explaining how genetic variants that enhance early-life fitness persist despite increasing susceptibility to age-related diseases [26,45]. Cancer and neurodegeneration represent divergent outcomes of this principle, arising from shared, evolutionarily conserved molecular pathways whose effects differ across cell types [46]. Traits favored for growth, reproduction, immune defense, or metabolic efficiency confer advantages in proliferative tissues but impose delayed detrimental consequences on PM neurons, thereby linking reproductive success with late-life vulnerability to both oncogenic transformation and neurodegeneration [47] (Figure 2).

#### 2.2.1. Huntingtin (HTT): Reproductive Fitness, Cancer Protection, and Neurodegeneration

CAG trinucleotide repeat expansion in the HTT gene causes HD. This leads to an elongated polyglutamine tract in mutant huntingtin protein (mHTT), leading to progressive neurodegeneration primarily in striatal projection neurons [48]. Despite this late-life neurotoxicity, HD-associated alleles endure in human populations, suggesting compensatory evolutionary advantages [49]. Epidemiological data indicate enhanced reproductive fitness and reduced early-onset cancer incidence among HD individuals [50]. Supporting this, Morton et al. developed a transgenic mouse model (R6/2_50) with 50 CAG repeats, which displayed hallmark HD pathology but also extended lifespan, improved locomotor function, increased reproductive success, and enhanced male survival compared to wild-type controls [51]. Huntingtin’s roles in vesicular trafficking, transcriptional regulation, and stress responses underpin cellular resilience during development and reproduction, yet these same features predispose neurons to proteostatic failure and aggregation with aging [51]. These findings suggest that HTT alleles confer developmental and reproductive benefits, while also lowering cancer risk and increasing susceptibility to late-onset neurodegeneration.

#### 2.2.2. APOE ε4: Infection Resistance, Metabolic Advantage, and Alzheimer’s Disease Risk

The APOE ε4 variant stands as the primary genetic factor increasing the risk for late-onset AD, increasing susceptibility by two- to twelvefold depending on zygosity and hastening disease onset [52]. Despite its substantial neurodegenerative impact in later life, APOE ε4 remains common in human populations, implying it provided important evolutionary benefits under ancestral conditions [52]. Anthropological and population studies suggest APOE ε4 was positively selected in environments with high pathogen exposure and nutritional challenges [53,54]. Trumble et al. found that carriers of APOE ε4 in parasite-dense settings experienced fewer diarrheal diseases, better growth, and enhanced cognitive function compared to non-carriers [55]. These advantages likely stem from improved lipid transport, more efficient cholesterol absorption, and stronger immune responses, all supporting survival and reproductive success during early life [56].

In contrast, in modern environments characterized by low pathogen burden and longer lifespan, these same immune-metabolic traits become detrimental. APOE ε4’s association with increased inflammatory signaling, disrupted lipid balance, and elevated amyloidogenic processing accelerates synaptic damage and neurodegeneration, illustrating antagonistic pleiotropy where early-life benefits contribute to late-life disease risk [57,58,59]. Beyond neurodegeneration, evidence shows APOE variants also influence cancer susceptibility and progression in a context-dependent manner [60]. Although APOE ε4 is best studied in AD, it acts as a low-penetrance genetic modifier in various cancers [61].

Meta-analyses indicate APOE ε4 is linked to higher breast cancer risk, primarily among Asian populations, though this association is inconsistent in Caucasians [62]. Additionally, APOE genotype affects cognitive outcomes in postmenopausal women diagnosed with early-stage breast cancer, especially after chemotherapy, suggesting a role in cancer-related cognitive vulnerability [63]. In gastric cancer, risk appears more strongly connected to the APOE ε2 allele, possibly through altered lipid metabolism, while APOE ε4’s role remains unclear [64].

More broadly, pan-cancer analyses demonstrate that APOE expression correlates with tumor growth, metastasis, immune infiltration, and patient prognosis across multiple cancer types [65]. These findings position APOE as a major switch of tumor-immune interactions and metabolic adaptation within the tumor microenvironment, with implications for chemotherapy and immunotherapy responses. However, the specific influence of APOE ε4 in these processes—beyond breast cancer—remains insufficiently understood and requires further study.

Collectively, APOE ε4 functions as a context-dependent immune-metabolic modifier, whose early-life advantages contrast with increased risk of neurodegeneration and cancer in aging populations.

#### 2.2.3. Androgen Receptor (AR) CAG Repeats: Balancing Reproductive Fitness and Oncogenic Risk

AR serves as a prime example of antagonistic pleiotropy, influencing both reproductive and somatic tissues [26]. The AR gene harbors a polymorphic CAG trinucleotide repeat within its transactivation domain, where repeat length inversely affects receptor transcriptional activity. Shorter CAG repeats enhance AR signaling and androgen sensitivity, while longer repeats diminish AR transactivation [26,66]. This genetic variation has been shaped by evolutionary forces favoring reproductive success but entails delayed oncogenic consequences in hormone-responsive tissues.

From an evolutionary standpoint, shorter AR CAG repeats confer significant early-life advantages. In males, increased AR activity promotes spermatogenesis, sperm viability, and androgen-dependent sexual function, thereby enhancing reproductive fitness [66,67,68]. Additionally, shorter repeats are linked to a lower risk of Kennedy’s disease, a neuromuscular disorder caused by excessive CAG expansions in AR [67]. In females, elevated AR activity associated with shorter repeats supports follicular development, oocyte viability, and reduces premature ovarian failure risk [69]. These benefits likely drove the positive selection and persistence of shorter AR CAG alleles in human populations.

However, the reproductive advantages conferred by heightened androgenic signaling come with substantial late-life costs. In proliferative, hormone-sensitive tissues, increased AR activity driven by shorter CAG repeats stimulates epithelial cell proliferation, elevating the risk of oncogenic transformation. Prostate cancer studies consistently link shorter AR CAG repeats with higher cancer risk, earlier onset, and more aggressive disease, including metastasis [70,71,72]. Although population variability exists, heightened AR signaling generally correlates with poorer prognosis and tumor aggressiveness [73]. Notably, short CAG repeats are less common in benign prostatic hyperplasia than in prostate cancer, suggesting distinct androgen-dependent mechanisms underlie benign versus malignant growth [72].

In breast cancer, AR CAG repeat length similarly modulates tumor behavior. Shorter repeats, indicative of increased AR activity, are associated with more aggressive tumors and poorer survival, whereas longer repeats are more frequent in less aggressive cases, implying a protective effect dependent on context [74]. Intriguingly, among BRCA1 mutation carriers, shorter AR CAG repeats correlate with reduced breast cancer risk, highlighting the complex interaction between AR signaling and tumor suppressor pathways [75]. These findings emphasize that AR-mediated trade-offs vary according to genetic background, tissue type, and hormonal milieu.

Conversely, ovarian cancer risk appears more strongly linked to longer AR CAG repeats. Reduced AR transactivation associated with longer repeats (≥22) may increase susceptibility, suggesting that diminished androgen signaling can also promote oncogenesis in certain cellular contexts [76]. This contrast underscores how the same polymorphism can yield divergent disease outcomes depending on tissue and endocrine environment.

Overall, AR CAG repeat polymorphisms enhance androgen signaling and reproductive fitness early in life while increasing cancer risk later. Conversely, longer repeats reduce AR activity and elevate ovarian cancer susceptibility depending on genetic, tissue, and hormonal context, reflecting evolutionary trade-offs linking fertility and oncogenic vulnerability across diverse tissues.

##### Integrative Perspective

Together, these examples illustrate how conserved regulatory pathways controlling proteostasis, immune–metabolic function, and hormone signaling produce tissue- and lifespan-specific effects. Genetic variants that enhance development and reproduction differentially influence cellular survival mechanisms in neurons versus proliferative tissues, shaping susceptibility to protein aggregation–related degeneration or malignant transformation. Recognizing these regulatory differences supports therapeutic strategies that emphasize tissue- and age-specific pathway modulation rather than uniform inhibition.

### 2.3. Selective Vulnerability: Evolutionary Outcome in Cancer and Neurodegeneration

Selective vulnerability denotes the disproportionate susceptibility of specific cell populations to disease, despite widespread exposure to genetic risks or pathological factors [77,78]. In both cancer and neurodegenerative disorders, this phenomenon reflects trade-offs embedded within conserved cellular programs rather than random cellular fragility. Although the affected cell types and underlying mechanisms differ, selective vulnerability in both contexts arises from evolutionary optimization of growth, repair, and function during early life, often at the expense of long-term resilience.

#### 2.3.1. Selective Vulnerability in Cancer: Stem Cells, Division, and Tissue Context

In cancer, selective vulnerability is closely tied to tissue-resident stem cells and their division dynamics [77]. The stem-cell division hypothesis posits that cancers emerge from normal tissue stem cells or their immediate progeny due to dysregulation of tightly regulated self-renewal processes [79]. Because stem cells sustain tissue maintenance through repeated divisions, they are inherently exposed to replication-associated mutations. Consequently, tissues with higher stem cell turnover accumulate oncogenic mutations more rapidly, explaining tissue-specific differences in cancer incidence [80].

This vulnerability is shaped by the stem cell niche, which regulates proliferation, differentiation, and quiescence [81]. Disruption of these signals—whether through genetic mutations or microenvironmental changes—can generate cancer stem cells (CSCs) that acquire self-sufficient proliferative capacity, driving tumor initiation, heterogeneity, and progression [80]. Because niche composition and signaling vary across tissues, CSC behavior and cancer susceptibility also differ accordingly [82].

Tissue-specific examples underscore this principle. In breast tissue, mammary stem and progenitor cells are tightly regulated during development and hormonal cycling. Dysregulation, particularly via oncogenic drivers like HER2 amplification, expands stem-like cell populations and promotes invasion and tumorigenesis [83,84]. In the liver, hepatocellular carcinoma can emerge from hepatic progenitor cells, through aberrant activation of conserved developmental pathways such as Wnt/β-catenin, Notch, Hedgehog, and TGF-β, highlighting how tissue-specific signaling environments shape selective cancer vulnerability [85,86].

Thus, selective vulnerability in cancer emerges from regenerative capacity and sustained stem-cell division within proliferative tissues.

#### 2.3.2. Selective Neuronal Vulnerability in Neurodegeneration: Post-Mitotic Costs of Optimization

By contrast, selective vulnerability in neurodegeneration targets PM neurons—cells that evolved to suppress proliferation to minimize cancer risk. Despite widespread expression of pathological proteins or genetic risk factors, only specific neuronal populations degenerate in diseases such as AD, PD, and ALS [87,88]. This selective neuronal loss underpins regional pathology and clinical heterogeneity.

Rather than random damage, selective neuronal vulnerability is increasingly viewed as a consequence of evolutionary optimization. The human brain evolved under pressures favoring cognitive efficiency, metabolic restraint, and functional longevity sufficient for reproduction and kin survival—not indefinite neuronal maintenance [89,90,91]. Consequently, neuronal systems conferring high adaptive value early in life are disproportionately vulnerable to degeneration in later decades.

Highly specialized neuronal populations—such as hippocampal excitatory neurons, substantia nigra dopaminergic neurons, and long-range cortical projection neurons—share features shaped by evolutionary pressures: extreme metabolic demand, extensive axonal arbors, dense synaptic connectivity, and sustained calcium signaling [92,93]. These properties support complex functions like learning, memory, and motor control but impose substantial energetic costs. Over time, high mitochondrial load and intense synaptic activity generate oxidative stress and metabolic by-products that exceed the declining proteostatic and autophagic capacity of aging neurons [94].

The PM state further shapes neuronal susceptibility. The suppression of cell division provides strong cancer protection—a major evolutionary advantage in long-lived species—but eliminates neuronal replacement capacity [8,27]. Genome surveillance mechanisms, including persistent p53 activation and strict cell-cycle checkpoints, protect neurons from oncogenic transformation but simultaneously sensitize them to apoptosis under cumulative stress [95,96]. Unlike proliferative tissues, where damaged cells can be replaced or diluted, neurons accumulate damage over decades [97,98].

Molecular specialization contributes additional vulnerability. Proteins such as α-synuclein and tau possess intrinsically disordered structures enabling rapid vesicle trafficking and cytoskeletal plasticity essential for synaptic function and learning [99,100]. However, these same features increase aggregation propensity as proteostasis declines with age [101,102]. Similarly, dopaminergic neurons undergo high cytosolic dopamine turnover, generating reactive species that damage proteins, lipids, and DNA—an evolutionary cost of systems optimized for reward processing and motor control [103,104].

Thus, neuronal selective vulnerability reflects the interaction between long-term metabolic demand, proteostatic burden, and limited regenerative capacity within specialized PM systems.

#### Converging Insights: Selective Vulnerability and Disease Risk

Despite differing cellular contexts, selective vulnerability in cancer and neurodegeneration shares dependence on the cellular microenvironment. In cancer, stem cell niches influence CSC emergence and tumor progression [80]. In neurodegeneration, region-specific microenvironments—defined by glial interactions, vascular support, metal homeostasis, and local proteostatic capacity—modulate neuronal susceptibility to protein misfolding and stress, as seen in prion diseases [102].

Across tissues, disease risk reflects how specialized cellular programs respond to cumulative stress within their structural and functional constraints. In proliferative systems, regenerative capacity increases opportunities for oncogenic mutation and clonal expansion. In contrast, PM neural systems optimized for long-term function rather than renewal become more vulnerable to metabolic and proteostatic decline.

### 2.4. Immune Trade-Offs: Linking Cancer and Neurodegeneration

The immune system illustrates how conserved regulatory networks influence disease susceptibility across tissues and lifespan. Immune responses, conserved across species, evolved under strong selective pressures to defend against pathogens, clear damaged cells, and suppress malignant transformation during early life, thereby maximizing survival and reproductive fitness [105]. However, as lifespan extends beyond evolutionary norms, these same immune mechanisms can become maladaptive, facilitating tumor immune evasion while driving chronic neuroinflammation and neurodegeneration [106,107]. Consequently, cancer and neurodegenerative diseases represent divergent outcomes of shared immune regulatory pathways functioning in distinct cellular and tissue contexts.

#### 2.4.1. Immune Trade-Offs in Cancer: Surveillance Versus Evasion

In cancer, immune dynamics revolve around the equilibrium between immune tolerance and immune surveillance [108]. While effective immune surveillance detects and eliminates emerging malignant cells, immune tolerance prevents autoimmunity against self-antigens. Tumors exploit this balance by hijacking conserved immune pathways to suppress antitumor immunity without compromising host viability [109]. Genetic alterations impairing antigen presentation—such as mutations in human leukocyte antigen (HLA) genes—and dysregulation of immune checkpoint signaling diminish cytotoxic T-cell recognition, enabling malignant cells to escape immune destruction [110].

Tumors also co-opt innate immune programs originally evolved for antiviral and stress responses. For instance, interferon-γ (IFN-γ), a critical cytokine in pathogen defense, is repurposed by tumors to upregulate non-classical MHC class I molecules like HLA-E (Qa-1b in mice), thereby suppressing the activity of natural killer (NK) cells and CD8^+^ T-cells [111]. Additional tumor strategies include expression of vascular cell adhesion molecule-1 (VCAM-1) to restrict T-cell infiltration and secretion of immunosuppressive factors such as galectin-1, which skew immune responses toward tolerance and tumor progression [112,113].

At the systemic level, immune tolerance further limits antitumor immunity. Many tumor antigens are self or developmentally regulated proteins, so central and peripheral tolerance mechanisms restrict the diversity and efficacy of tumor-reactive T cells [114]. This reflects an evolutionary compromise: immune systems optimized to prevent autoimmunity inherently reduce long-term cancer surveillance. Thus, tumor immune escape is not immune failure but a predictable consequence of immune systems prioritizing organismal integrity during early life.

#### 2.4.2. Neuroimmune Trade-Offs in Neurodegeneration: Protection Versus Toxicity

In the nervous system, immune trade-offs manifest differently. Brain innate immune responses evolved to clear debris, combat infection, and refine neural circuits during development. However, chronic stimulation of these pathways with aging leads to persistent neuroinflammation, synaptic loss, and neuronal death. Disorders such as AD and PD illustrate how sustained immune activation can become pathogenic in long-lived neural tissue [115,116].

Microglia exemplify this shift. Originating from embryonic myeloid progenitors, microglia rapidly detect danger signals, phagocytose debris, and sculpt synaptic networks via complement-mediated pruning—functions essential in pathogen-rich ancestral environments and neurodevelopment [117,118]. With aging, microglia adopt a “primed” phenotype characterized by heightened inflammatory responses, reduced phagocytic capacity, and sustained cytokine secretion [119]. IFN-γ further sensitizes aged microglia, exacerbating oxidative stress and neuronal injury [120]. Thus, immune mechanisms that once preserved neural integrity become drivers of neurodegeneration in later life.

Genetic regulators such as TREM2 highlight the shared immune logic linking cancer and neurodegeneration. TREM2 signaling promotes myeloid cell survival, lipid metabolism, and immune regulation—traits advantageous for host defense early in life. In aging, altered TREM2 function modifies microglial inflammatory states, influencing AD risk and immune suppression within tumor microenvironments [121]. These parallels underscore how conserved immune regulators can contribute to distinct pathological outcomes depending on tissue context.

#### 2.4.3. Glial Evolution and Immune Specialization

The expansion and specialization of glial populations further shape immune vulnerability in the brain. Astrocytes and oligodendrocytes evolved beyond structural support to regulate synaptic transmission, metabolic coupling, blood–brain barrier integrity, and immune signaling. Human astrocytes are larger and more complex than those in non-primates, supporting advanced cognition but increasing vulnerability to metabolic and inflammatory stress [122,123]. Under chronic injury or aging, astrocytes adopt reactive phenotypes that impair glutamate clearance, disrupt ionic homeostasis, and secrete complement factors promoting synaptic elimination [124,125].

Oligodendrocytes present a parallel compromise. Although myelination enhances conduction velocity and cognitive integration, it imposes high metabolic demands reliant on lipid synthesis and mitochondrial function. Oligodendrocyte lineage cells are highly vulnerable to oxidative and inflammatory stress, and immune-metabolic dysregulation impairs remyelination, contributing to neurodegenerative vulnerability [126,127].

#### 2.4.4. The Neuro–Immune–Cancer Axis: Systemic Trade-Offs in Regulation

Beyond cell-intrinsic immune mechanisms, cancer and neurodegeneration emerge from systemic neuro–immune crosstalk sculpted by evolution. The nervous system regulates immune responses through conserved neuroimmune and neuroendocrine signaling pathways. Neurotransmitters, neuropeptides, and neurotrophic factors play crucial roles in tailoring immune cell functions by influencing the behavior of T cells, macrophages, dendritic cells, and myeloid-derived suppressor cells (MDSCs), thereby shaping immune surveillance, inflammation, and tumor progression [128].

This coordination occurs within the neuro-endocrine–immune axis, optimized to balance rapid immune activation with suppression of chronic inflammation. Neural and endocrine signals influence immune tone, metabolism, angiogenesis, and cellular proliferation, affecting tumor growth, invasion, and therapeutic resistance [129]. Emerging evidence indicates that neurodegenerative processes remodel tumor immune microenvironments by altering immune infiltration and cytokine profiles, while tumor-driven immune reprogramming exacerbates neural dysfunction [128]. This bidirectional interaction reinforces the context-dependent relationship between cancer and neurodegeneration.

##### Convergent Immune Logic Across Diseases

Despite divergent outcomes, cancer and neurodegeneration share common immune principles governed by conserved signaling pathways such as NF-κB, JAK/STAT, PI3K/AKT, Wnt/β-catenin, TGF-β, and Hippo. In cancer, these pathways promote immune evasion, stromal remodeling, and angiogenesis; in neurodegeneration, they drive sterile inflammation, synaptic loss, and neuronal death [130,131].

The differing outcomes reflect tissue-specific regulation rather than fundamentally distinct immune systems. In proliferative environments, immune modulation influences clonal expansion and tumor persistence. In the brain, sustained inflammatory signaling contributes to progressive neuronal dysfunction. Recognizing these shared regulatory networks supports therapeutic strategies that reshape immune responses in a tissue- and age-specific manner rather than broadly amplifying or suppressing immunity.

## 3. Beyond Peto’s Paradox: Lessons in Disease Resistance from Unusual Species

Multicellular organisms face the evolutionary challenge of maintaining tissue integrity while preventing unchecked cell proliferation over extended lifespans [132]. Traditional models predict that cancer risk should correlate positively with body size and lifespan, as larger and longer-lived species accumulate more cells and undergo more replication events, elevating the chance of oncogenic mutations [133,134]. Yet, comparative epidemiological data contradict this, revealing no direct correlation between cancer incidence and these factors across species—a phenomenon called Peto’s paradox. This paradox underscores the evolution of potent, species-specific mechanisms that uncouple cancer risk from organismal size and lifespan [133,135].

**Figure 3:**
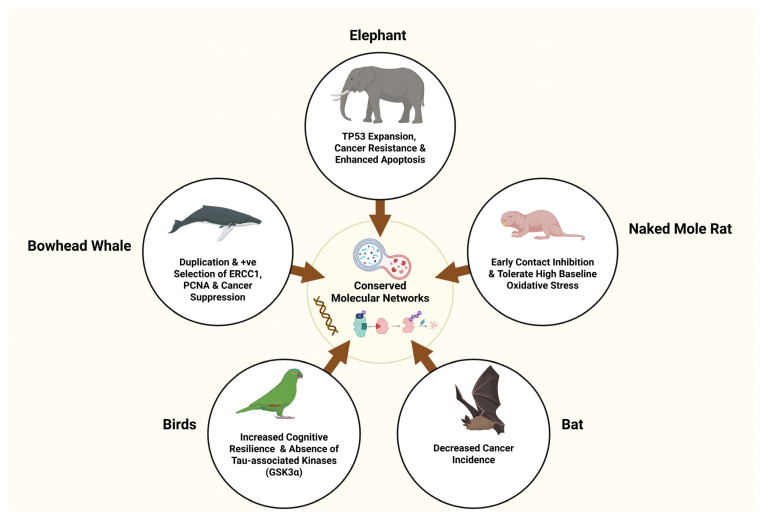
Conserved Pathways and Species-Specific Tuning in Natural Disease Resistance.

Beyond cancer, Peto’s paradox highlights a broader biological balance. Species achieving exceptional longevity or gigantism must suppress malignant transformation while preserving long-term cellular function, especially in PM tissues like the nervous system. Comparative biology thus provides a framework to understand how evolution tunes conserved molecular pathways—governing genome stability, proteostasis, metabolism, and immune regulation—to balance tumor suppression with neural maintenance. These natural adaptations clarify why identical pathways in humans can lead to divergent outcomes: uncontrolled cell division in cancer versus gradual neuronal loss in neurodegeneration (Figure 3).

### 3.1. Genome Surveillance and Growth Control as Foundations of Disease Resistance

Long-lived and large-bodied mammals exemplify how reinforced genome surveillance and growth control underpin both cancer resistance and tissue preservation.

Elephants (*Loxodonta africana* and *Elephas maximus*) offer a striking example. Despite their vast cellular abundance, elephants display markedly lower cancer mortality. Genomic studies reveal an expanded tumor suppressor gene repertoire, particularly at least 20 copies of TP53, which increase DNA damage sensitivity and lower the apoptotic threshold [136,137]. This mechanism promotes early removal of damaged or potentially malignant cells, favoring organismal integrity over individual cell survival.

Crucially, this intensified tumor suppression does not impair neural function. Elephants maintain complex cognition, long-term social memory, and learning over decades. Comparative analyses suggest that enhanced DNA repair capacity, antioxidant defenses, and tightly regulated neuroimmune signaling preserve neural circuits while preventing chronic inflammation [138,139]. Thus, elephants demonstrate that robust genome surveillance can coexist with long-term neural maintenance when precisely regulated.

Cetaceans extend this principle to extreme longevity. The bowhead whale (Balaena mysticetus), the longest-lived mammal, surpasses 200 years of age. Comparative genomic and transcriptomic analyses reveal lineage-specific adaptations in DNA repair, regulation of cell cycle, and genome stability, including positive selection and duplication of genes such as ERCC1 and PCNA [140,141]. These adaptations reinforce cancer suppression and promote long-term tissue preservation. Sustained expression of DNA repair and checkpoint genes likely limits mutation accumulation while avoiding excessive apoptosis that could damage PM tissues.

### 3.2. Proteostasis and Metabolic Stability as Shared Defenses Against Cancer and Neurodegeneration

Enhanced proteostasis and metabolic resilience emerge as convergent strategies across taxa that counter both malignant transformation and neurodegenerative decline.

Bowhead whales exhibit sustained upregulation of the ubiquitin–proteasome system and autophagy pathways across tissues [142]. These mechanisms facilitate efficient clearance of damaged or misfolded proteins, mitigating oncogenic stress and preventing accumulation of aggregation-prone species central to NDDs such as AD and PD. Although direct neuropathological data remain limited, cetacean molecular profiles align with resistance to proteostasis collapse, mitochondrial dysfunction, and chronic inflammation—key contributors to both cancer progression and neurodegeneration in humans.

The naked mole-rat (*Heterocephalus glaber*) represents a distinct but complementary evolutionary strategy. Despite its small size, it exhibits exceptional longevity, near-complete cancer resistance, and preserved neural function. Rather than relying predominantly on apoptosis, naked mole-rats enforce stringent tissue-level growth control via early contact inhibition facilitated by ultra-high-molecular-weight hyaluronan and a two-tiered cell-cycle arrest involving p27 and p16 [143,144]. Concurrently, they maintain robust proteostasis through sustained autophagic and proteasomal activity, coupled with dampened inflammatory responses [145,146].

Remarkably, naked mole-rats tolerate high baseline oxidative stress without neuronal loss, suggesting an evolutionary shift from damage avoidance to damage tolerance. This strategy preserves long-term tissue function while limiting chronic inflammation and proteotoxicity [147]. This tolerance suppresses tumor-promoting inflammation and neuroimmune priming, linking cancer resistance with neuroprotection.

### 3.3. Neural Resilience and Cognitive Longevity Across Vertebrates

Genome surveillance is fundamental to disease resistance; however, maintaining neural function requires additional mechanisms beyond apoptosis and cell renewal. This presents a distinct evolutionary challenge, particularly in species with prolonged lifespans or heightened cognitive demands.

Both elephants and cetaceans display molecular signatures consistent with sustained neural preservation, including enhanced mitochondrial maintenance, antioxidant defenses, and regulated glial activation [139,140,142]. These features mitigate age-associated protein aggregation, metabolic failure, and sterile inflammation: processes central to human neurodegenerative diseases.

Long-lived, cognitively sophisticated birds such as corvids and parrots exemplify convergent neural resilience beyond mammals. Despite high neuronal density, extended lifespans, and advanced cognitive abilities—including problem-solving, tool use, and long-term memory—these species exhibit low natural incidence of neurodegeneration [148,149]. Their genomes harbor distinctive features relevant to neurodegenerative resistance, including the absence of tau-associated kinases like GSK3α and refined proteostasis regulation [150]. These adaptations may reduce susceptibility to pathological protein aggregation while sustaining lifelong learning and synaptic plasticity [151]. Moreover, birds appear inherently resistant to virus-driven tumorigenesis. For example, avian polyomaviruses, which induce inflammatory and proliferative disorders in parrots, have not been documented to cause tumors in their natural hosts. This contrasts with mammalian polyomaviruses, which are oncogenic in experimental rodent models [152]. Such observations imply that avian species possess distinct oncogenic viral response mechanisms, potentially involving more effective immune surveillance or unique cellular growth control pathways. Collectively, these findings reinforce the concept that evolutionarily conserved molecular networks can be selectively modulated across taxa to concurrently suppress neurodegeneration and oncogenesis.

Bats further challenge life-history expectations. Despite small body size and the extreme metabolic demands of flight, many bat species are exceptionally long-lived and exhibit low cancer incidence. Genomic studies reveal adaptations in DNA repair, cell-cycle control, and innate immune signaling, including dampened inflammasome activation and constitutive interferon signaling [153,154,155]. This immune profile enables efficient pathogen defense while limiting chronic inflammation, suppressing tumorigenesis without provoking neuroinflammatory damage. Like naked mole-rats, bats tolerate elevated oxidative stress, reinforcing damage tolerance as a recurring evolutionary strategy [156].

### 3.4. Mechanistic Insight from Short-Lived Models: Pathway Tuning over Lifespan

Resistance to cancer and neurodegeneration is not exclusive to long-lived species. Model organisms such as *Drosophila melanogaster* illustrate that selective tuning of conserved pathways, rather than lifespan alone, governs disease outcomes. *Drosophila* serves as an excellent model to study both cancer and neurodegeneration because it shares deeply conserved regulators of growth, proteostasis, mitochondrial quality control, and innate immunity with humans.

In cancer models, *Drosophila* studies have elucidated fundamental processes such as clonal evolution, tumor microenvironment interactions, and drug resistance. Critical tumor suppressor pathways like the Hippo pathway were first identified using *Drosophila* genetics [157]. Hippo signaling controls organ size and cell proliferation, balancing cell survival and apoptosis, which helps prevent uncontrolled growth [158]. Selective modulation of this pathway influences whether cells undergo normal growth control or tumorigenic overgrowth [159,160]. Additionally, *Drosophila* models reveal how regulation of cell polarity and intercellular cooperation affects tumor progression, highlighting that finely tuned signaling rather than a static lifespan limits cancer development [159].

In neurodegeneration, *Drosophila* models mimic human disorders such as Parkinson’s disease through genetic manipulation of conserved genes involved in synaptic function, oxidative stress, autophagy, and mitochondrial integrity [161]. For example, the Highwire ubiquitin ligase regulates axonal degeneration by modulating protein levels that protect neurons, illustrating pathway tuning that determines neuronal survival or degeneration [161,162]. Hippo signaling also plays a role in neurodegeneration by controlling apoptosis; its dysregulation can lead to neural cell loss, and its targeted inactivation rescues neurons from degeneration, indicating the importance of pathway balance [163]. Furthermore, common pathogenic mechanisms involve apoptosis, protein misfolding, and histone acetylation, with modifiers identified in fly models that modify toxicity in both retinal and brain neurons, underscoring the conserved molecular determinants of neurodegeneration [164,165,166].

Thus, outcomes in disease—cancer versus neurodegeneration—depend on the selective and tissue-specific tuning of conserved signaling pathways such as Hippo, ubiquitin ligase networks, autophagy, and apoptosis regulators. These pathways govern cellular growth, survival, and clearance mechanisms and act independently of the organism’s lifespan to influence disease susceptibility and progression in *Drosophila*. This highlights that resistance to cancer and neurodegeneration arises from precise regulatory control of conserved molecular pathways rather than longevity alone.

### 3.5. Evolutionary Principles Emerging from Natural Disease Resistance

Across diverse taxa—elephants, whales, naked mole-rats, bats, birds, and *Drosophila*—species resistant to both cancer and neurodegeneration converge on shared evolutionary priorities: reinforced genome stability, robust proteostasis, restrained inflammation, metabolic resilience, and precise regulation of growth and stress-response pathways [167]. These strategies reflect life-history optimizations for early-life survival, reproductive success, and long-term tissue maintenance rather than disease-specific adaptations.

Importantly, these models demonstrate that cancer suppression and neuroprotection are not inherently opposing outcomes but depend on how conserved pathways are tuned across tissues and lifespan.

By elucidating how evolution resolves these competing demands, cross-species comparisons provide a conceptual bridge to the following section, which examines how conserved molecular pathways—shared across species and cell types—produce divergent outcomes in cancer and neurodegeneration depending on cellular identity, regulatory state, and timing. (Supplementary Table S1 encapsulates these convergent evolutionary mechanisms underlying natural resistance to neurodegeneration and cancer, highlighting representative species, key features, and disease-relevant outcomes).

## 4. Context-Dependent Outcomes of Conserved Molecular Pathways in Cancer and Neurodegeneration

**Figure 4:**
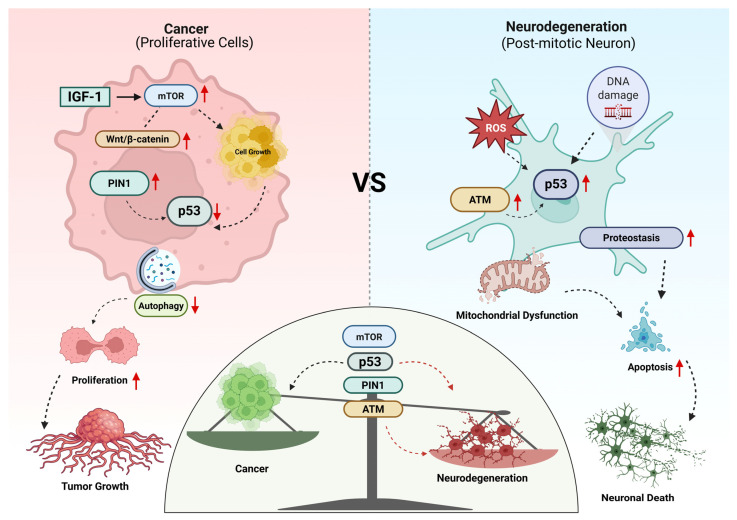
Context-Dependent Divergence of Conserved Molecular Pathways in Cancer and Neurodegeneration. Solid arrows indicate signaling relationships, dashed arrows represent indirect downstream effects, and red arrows denote changes in pathway activity (upward arrows indicate increased activity, whereas downward arrows indicate decreased activity).

Cancer and neurodegeneration both stem from dysregulation of deeply conserved molecular systems rather than distinct disease-specific pathways. Core cellular programs regulating genome integrity, mitochondrial function, proteostasis, developmental signaling, metabolism, and immune responses are shared across tissues and species. However, the biological consequences diverge sharply based on cellular identity, proliferative ability, metabolic demands, and the duration of signaling. In some contexts, these pathways support survival, clonal expansion, and tissue remodeling, whereas in others, similar perturbations lead to synaptic failure, mitochondrial dysfunction, and irreversible cell loss [1,10,11,19] (Figure 4). This section integrates mechanisms to explain how conserved molecular networks drive opposing disease trajectories in cancer and neurodegeneration.

### 4.1. Genome Surveillance and DNA Damage Responses: Protection Versus Attrition

Genome surveillance integrates DNA repair, cell-cycle checkpoint enforcement, and apoptosis via a conserved network centered on p53, PIN1, and ATM. Rather than functioning as isolated tumor suppressors, these molecules form a dynamic stress-response network whose outcomes depend on cellular identity, proliferative capacity, and metabolic state.

In proliferative cells, transient activation of p53 induces DNA repair and cell-cycle arrest through effectors such as p21, thereby preventing the propagation of damaged genomes [168,169]. When TP53 function is compromised, checkpoint control collapses, enabling proliferation despite genomic instability and facilitating oncogenic transformation [170]. In this context, p53 primarily functions as a barrier to clonal expansion of damaged cells.

In contrast, in PM neurons with limited regenerative capacity, sustained p53 activation shifts from repair toward apoptosis and mitochondrial dysfunction, contributing to synaptic degeneration [171]. Neuronal p53 dysregulation often arises from oxidative stress or aberrant interactions with misfolded proteins, converting a genome-protective response into a driver of progressive neurodegeneration [172]. Thus, a mechanism that constrains proliferation in dividing tissues can promote progressive attrition in long-lived neural populations.

PIN1 further refines this genome surveillance by inducing conformational switching at phosphorylated Ser/Thr–Pro residues and thus regulating the stability, activity, and interactions of numerous phosphorylated proteins, including p53 and key cell-cycle regulators [173,174]. This fine-tuning of protein dynamics governs cell fate decisions in response to genomic stress [173].

In cancer, PIN1 is frequently overexpressed or hyperactivated. It stabilizes proliferative and survival-promoting substrates such as cyclin D1, c-Myc, and mutant p53, amplifying oncogenic signaling, tumor progression and therapeutic resistance [175]. Notably, PIN1-mediated enhancement of p53 activity can paradoxically support tumor growth when p53 is mutated or dysfunctional, underscoring its context-dependent oncogenic potential [176].

In PM neurons, however, PIN1 levels and activity decline with aging and disease. Reduced PIN1 activity has been associated with tau hyperphosphorylation and increased amyloidogenic processing of amyloid precursor protein, linking impaired prolyl isomerization to proteostasis failure in AD [177]. This breakdown of PIN1-mediated conformational control links genome surveillance failure directly to proteostasis collapse, exacerbating neurodegeneration.

Nevertheless, the role of PIN1 in neurodegeneration remains incompletely resolved. While several studies support a protective function whereby PIN1 loss accelerates tau pathology, others propose that altered PIN1 expression may represent a compensatory response to proteotoxic stress rather than a primary driver of disease. Whether PIN1 dysregulation is causative, permissive, or reactive in neurodegenerative progression therefore remains debated.

Upstream, ATM kinase serves as a central kinase that senses DNA double-strand breaks, orchestrating DNA repair, checkpoint activation, and apoptosis via phosphorylation of substrates including p53, CHK2, and histone variant H2AX [177]. In proliferative cells, ATM enforces genomic fidelity by coordinating repair pathways and limiting mutation accumulation, functioning as a critical tumor suppressor [178].

Beyond its nuclear functions, ATM also regulates mitochondrial dynamics, redox balance, and synaptic stability [179,180]. ATM deficiency, as observed in ataxia-telangiectasia, results in increased cancer susceptibility alongside progressive neurodegeneration, exemplifying the dual consequences of impaired DNA damage responses [181,182]. This phenotype illustrates how disruption of a single conserved pathway can simultaneously enhance proliferative risk and accelerate neuronal loss.

Evolutionary and comparative studies indicate that ATM’s dual functionality is conserved, shaped by selective pressures balancing genome surveillance with long-term tissue preservation [136,137]. This regulatory tuning sustains malignant transformation suppression alongside neural function preservation over extended lifespans [142,146].

Collectively, the p53–PIN1–ATM axis illustrates a central principle of conserved pathway biology: genome surveillance mechanisms are not inherently oncogenic or neurotoxic. Their consequences depend on activation dynamics, regenerative capacity, and metabolic context. In proliferative environments, this network constrains malignant transformation. In long-lived PM neurons, chronic or dysregulated activation can convert protective stress responses into progressive cellular attrition. The divergence reflects differences in regenerative capacity, metabolic demand, and activation dynamics rather than differences in pathway identity.

### 4.2. Mitochondrial Quality Control and Redox Signaling: Adaptive Flexibility Versus Energetic Collapse

Mitochondria serve as central hubs for cellular metabolism, redox homeostasis, and apoptotic signaling [183]. Their integrity is maintained by evolutionarily conserved regulators that coordinate mitochondrial dynamics, selective autophagic clearance (mitophagy), and antioxidant defenses [184]. Key molecules in these processes include the PINK1–Parkin pathway, Voltage-Dependent Anion Channel 1 (VDAC1), and the transcription factor NRF2 [185,186,187].

#### 4.2.1. PINK1–Parkin-Mediated Mitophagy

The PINK1–Parkin axis selectively removes damaged mitochondria to avert accumulation of damaged organelles and limit ROS production [188,189]. Upon mitochondrial depolarization, PINK1 accumulates on the outer membrane and recruits Parkin, which ubiquitinates mitochondrial substrates and targets them for autophagic degradation [190]. This pathway maintains mitochondrial turnover and metabolic adaptability.

Despite its well-characterized molecular framework, the extent to which PINK1–Parkin-mediated mitophagy governs basal mitochondrial quality control in vivo remains debated. While robust in cultured systems, neuronal mitophagy in physiological settings appears less dependent on Parkin, and alternative receptor-mediated pathways (e.g., BNIP3, NIX, FUNDC1) may predominate in certain tissues. These discrepancies highlight unresolved questions regarding tissue specificity, compensatory mechanisms, and stress-dependent activation of parallel mitophagy programs.

In neurodegeneration, particularly PD, mutations in PINK1 or Parkin impair mitochondrial clearance, contributing to oxidative stress and dopaminergic vulnerability [191]. Although genetic and cellular models support a neuroprotective role for this pathway, causal links between mitophagy failure and neuronal loss in mammalian systems remain incompletely resolved and may depend on additional stressors or compensatory pathways.

In cancer, the impact of mitophagy varies with tumor context: it represses tumor initiation by limiting ROS-induced DNA damage but also sustains tumor survival under hypoxic or metabolic stress by maintaining mitochondrial fitness [192]. Conversely, in established tumors, mitophagy can facilitate survival under hypoxia or therapeutic stress by preserving mitochondrial function and limiting apoptosis. Beyond organelle clearance, Parkin also modulates non-mitochondrial substrates and transcriptional programs, including NF-κB signaling, thereby influencing inflammatory and survival networks in tumor cells.

These opposing roles of PINK1–parkin-mediated mitophagy illustrate how a conserved quality control pathway can both constrain tumor initiation and promote tumor adaptation, depending on disease stage and metabolic context. Its impact on neuroprotection and tumor biology reflects regulatory context rather than intrinsic pathway identity.

#### 4.2.2. VDAC1 Functional Duality

VDAC1, a mitochondrial outer membrane channel, regulates metabolite and ion flux essential for production of energy and apoptosis [193]. By coupling mitochondrial metabolism to cellular survival pathways, VDAC1 functions as a metabolic and death-signaling checkpoint whose effects vary markedly across pathological contexts.

In cancer, VDAC1 is frequently overexpressed and supports tumor growth by sustaining metabolic reprogramming and resistance to apoptosis. A key mechanism involves its interaction with hexokinases (HK-I and HK-II), which are often upregulated in tumors. Binding of hexokinase to VDAC1 facilitates high glycolytic flux characteristic of the Warburg phenotype while simultaneously inhibiting cytochrome c release, thereby suppressing apoptosis and enhancing chemoresistance [194]. Disruption of VDAC1–hexokinase complexes restores apoptotic signaling in experimental systems, underscoring the importance of this interaction in tumor maintenance. Additionally, cytoskeletal components such as β-tubulin modulate VDAC1 permeability, influencing the balance between oxidative phosphorylation and glycolysis and further shaping tumor metabolic plasticity.

Paradoxically, VDAC1 can also mediate programmed cell death. Oligomerization of VDAC1 forms large pores that permit the release of apoptogenic factors, including cytochrome c, promoting mitochondria-dependent apoptosis. VDAC1 oligomerization has also been implicated in ferroptosis, linking mitochondrial permeability to iron-dependent oxidative cell death. Thus, while elevated VDAC1 expression supports tumor survival under certain conditions, its structural reorganization can instead promote tumor-suppressive cell death. The determinants governing this switch—expression level, post-translational modification, interacting partners, and metabolic state—remain incompletely defined and may vary across tumor types.

In neurodegeneration, VDAC1 exhibits a similarly complex duality. Under physiological conditions, it maintains neuronal survival by regulating mitochondrial metabolism, calcium flux, and ATP production. However, VDAC1 also acts as a docking platform for misfolded proteins associated with neurodegenerative diseases, including amyloid-β and tau in Alzheimer’s disease, α-synuclein in Parkinson’s disease, and mutant SOD1 in amyotrophic lateral sclerosis [195,196]. Binding of these pathogenic proteins alters VDAC1 conformation and permeability, disrupts bioenergetics, and promotes apoptotic signaling. In Alzheimer’s models, VDAC1 overexpression and hyperphosphorylation correlate with increased mitochondrial permeability and neuronal death, whereas loss of protective VDAC1–hexokinase interactions exacerbates apoptosis [197].

Regulatory mechanisms further complicate its role. Post-translational modifications such as phosphorylation and ubiquitination influence VDAC1 oligomerization and stability. For example, E3 ubiquitin ligases that promote VDAC1 degradation can protect dopaminergic neurons by limiting excessive mitochondrial permeability, suggesting that balanced VDAC1 turnover is neuroprotective. Conversely, excessive VDAC1 accumulation or oligomerization has been linked to neuronal loss in models of ischemia and polyglutamine expansion disorders.

Isoform-specific differences also remain under investigation. Although VDAC1 appears more strongly associated with pro-apoptotic signaling than other VDAC isoforms, the relative contributions of VDAC2 and VDAC3 to mitochondrial homeostasis and cell fate are not fully resolved. Whether VDAC1 upregulation is causative in neurodegeneration or represents a secondary response to mitochondrial stress remains debated.

Collectively, VDAC1 exemplifies how a conserved metabolic channel can support tumor survival, trigger tumor cell death, preserve neuronal function, or promote neurodegeneration depending on structural state, interacting partners, and cellular context. Its dual role in apoptosis, ferroptosis, and metabolic adaptation makes VDAC1 a promising yet high-risk therapeutic target, as indiscriminate inhibition could impair normal mitochondrial function while insufficient modulation may fail to shift the balance toward disease-modifying outcomes.

#### 4.2.3. NRF2 Signaling Complexity

NRF2 is a key regulator of cellular antioxidant responses, modulating gene expression to counteract oxidative stress [198]. Under basal conditions, NRF2 is retained in the cytoplasm and targeted for proteasomal degradation by its repressor KEAP1. Stress-induced modification of KEAP1 cysteine residues disrupts this interaction, allowing NRF2 stabilization, nuclear translocation, and transcriptional activation of cytoprotective programs [199]. Through regulation of glutathione synthesis, NADPH production, xenobiotic metabolism, and mitochondrial function, NRF2 maintains cellular resilience against metabolic and inflammatory stress.

In cancer, however, NRF2 exhibits a dual role. In normal or premalignant cells, NRF2 activation limits oxidative DNA damage and inflammation, thereby suppressing tumor initiation [200]. Consistent with this protective function, Nrf2-deficient mice show increased susceptibility to carcinogen-induced tumorigenesis. In contrast, in established tumors, constitutive NRF2 activation—often driven by mutations in KEAP1 or NRF2, epigenetic silencing of KEAP1, oncogenic signaling (e.g., KRAS), or metabolic reprogramming—confers a selective advantage. Persistent NRF2 activity enhances antioxidant buffering, supports metabolic flexibility, promotes glutathione-dependent detoxification, increases drug efflux transporter expression, and contributes to resistance against chemotherapy and radiotherapy. High NRF2 expression or KEAP1 mutation status frequently correlates with poor prognosis and reduced therapeutic responsiveness. Thus, NRF2 can function as a tumor suppressor during initiation but as a tumor-promoting factor during progression and treatment adaptation. The boundary between these roles remains incompletely defined and likely depends on tumor stage, mutational landscape, and metabolic context.

In neurodegenerative diseases, NRF2 is generally regarded as protective. Oxidative stress and mitochondrial dysfunction are early features of disorders such as AD and PD, and impaired NRF2 signaling has been associated with reduced antioxidant defenses and heightened neuronal vulnerability [201,202]. Activation of NRF2 in astrocytes, in particular, enhances redox support to neighboring neurons and mitigates inflammatory damage. Pharmacologic NRF2 inducers have shown neuroprotective effects in preclinical models, improving mitochondrial integrity, reducing proteotoxic stress, and attenuating neuroinflammation.

However, the neuroprotective paradigm is not without complexity. NRF2 activation appears more robust in glial cells than in neurons, raising questions about cell-type–specific responsiveness and therapeutic targeting. Moreover, chronic or excessive NRF2 activation may carry risks. Sustained NRF2 signaling has been associated with shortened lifespan in *Drosophila* and may theoretically increase oncogenic potential if prolonged systemically. Clinical experience with potent NRF2 activators, such as electrophilic compounds, has revealed off-target toxicities, underscoring the challenge of safely modulating this pathway.

Collectively, NRF2 exemplifies how a conserved stress-response pathway can generate divergent disease outcomes. Moderate, transient activation preserves genomic and metabolic integrity, whereas chronic or constitutive activation can enable tumor survival and therapy resistance. In the nervous system, reduced NRF2 activity contributes to oxidative injury, yet indiscriminate activation may carry long-term risks. Effective therapeutic strategies therefore require context-sensitive modulation—accounting for disease stage, tissue specificity, mutational background, and duration of activation—rather than uniform enhancement or inhibition of NRF2 signaling.

##### Integration of Mitochondrial and Redox Networks

The PINK1/Parkin pathway, VDAC1-mediated metabolic control, and NRF2-driven antioxidant responses form an interconnected axis that coordinates mitochondrial turnover, redox balance, and cell fate. Mitophagy limits ROS accumulation, NRF2 adjusts antioxidant capacity and metabolic gene expression, and VDAC1 integrates substrate flux with apoptotic signaling. Together, these systems determine whether cells adapt to stress or undergo programmed death.

In cancer, this network is frequently rewired to favor survival. Enhanced mitophagy preserves mitochondrial fitness under metabolic stress, NRF2 hyperactivation buffers ROS and promotes metabolic flexibility, and VDAC1–hexokinase interactions suppress apoptosis. These adaptations collectively support tumor resilience and therapy resistance.

In post-mitotic neurons, however, the same regulatory systems operate within a narrow tolerance window. Impaired mitophagy, dysregulated VDAC1 permeability, or insufficient NRF2 activation leads to cumulative mitochondrial dysfunction and oxidative injury. Importantly, excessive activation may also be harmful, underscoring the need for tightly balanced signaling.

Comparative studies of long-lived, cancer-resistant species suggest that longevity arises not from novel pathways but from refined regulation of existing mitochondrial and redox programs [142,146]. Yet the molecular determinants of this balance remain incompletely understood.

Thus, conserved mitochondrial–redox networks do not intrinsically promote cancer or neurodegeneration. Disease emerges from context, timing, and activation dynamics. Therapeutic strategies should therefore prioritize calibrated, tissue-specific modulation rather than global activation or inhibition.

### 4.3. Proteostasis Networks: Stress Adaptation Versus Proteotoxicity

Proteostasis—the balanced regulation of protein folding, trafficking, and degradation—is a fundamental network comprising molecular chaperones, the ubiquitin–proteasome system (UPS), and the autophagy–lysosomal pathway [203]. These systems collectively mitigate proteotoxic stress and preserve cellular function, yet their activation yields fundamentally divergent effects in cancer and neurodegeneration, reflecting differences in cellular context, metabolic demand, and regenerative capacity [204,205,206].

Heat shock proteins (HSP27, HSP70, HSP90) are central chaperones that maintain folding capacity under stress. Central to proteostasis, the molecular chaperone Hsp90 stabilizes a broad array of proteins engaged in cellular growth, survival, and stress responses [207]. In cancer, Hsp90 and its co-chaperone Aha1 are frequently upregulated, enhancing the maturation and persistence of oncogenic clients and conferring resistance to therapeutic stressors [208,209]. Yet clinical inhibition of Hsp90 has shown limited success in solid tumors, partly due to compensatory heat shock responses and systemic toxicity, underscoring the complexity of targeting chaperone networks. Conversely, in neurons, Hsp90–Aha1 complexes assist in managing aggregation-prone proteins such as tau, α-synuclein and TDP-43 [210,211,212]. While increased chaperone activity may initially mitigate aggregation, excessive stabilization of misfolded conformers can paradoxically preserve toxic oligomers. Co-chaperones such as BAG3 promote selective autophagy of aggregated proteins, yet dysregulated BAG3 activity may also reinforce survival pathways or alter cell death signaling. Whether chaperone upregulation in neurodegeneration is protective, maladaptive, or stage-dependent remains incompletely resolved.

Proteostasis is further supported by degradative pathways. The UPS, a highly conserved mechanism, selectively clears misfolded and regulatory proteins [213]. In cancer, dysregulation of ubiquitination and deubiquitination enzymes stabilizes cell-cycle and survival proteins, fostering oncogenesis and representing a therapeutic target for proteasome inhibitors, especially in hematologic malignancies [214,215]. For example, the E3 ligase STUB1/HSP70 complex regulates androgen receptor variant stability in advanced prostate cancer, directly influencing drug resistance. Proteasome inhibitors are effective in hematologic malignancies but have largely failed in solid tumors, reflecting tumor-specific proteostatic adaptations and systemic toxicity. In neurodegenerative diseases, partial UPS impairment contributes to accumulation of ubiquitinated protein aggregates, intensifying proteotoxic stress and neuronal loss [216,217]. Importantly, mammalian and *Drosophila* models demonstrate that the UPS remains functionally accessible, underscoring its therapeutic potential to enhance clearance of neurotoxic proteins [218,219].

Autophagy, alongside chaperone and proteasomal systems, facilitates lysosomal degradation of aggregated proteins and impaired organelles. During early tumorigenesis, basal autophagy inhibits malignant transformation by removing damaged organelles, reducing reactive oxygen species (ROS) accumulation, and maintaining genomic stability. Impaired autophagy has been shown to promote spontaneous tumor development in experimental models [220,221].

Conversely, established tumors exploit autophagy to survive hypoxia, nutrient deprivation, and therapeutic stress. This process provides recycled metabolites and enhances resistance to chemotherapy, radiotherapy, and immunotherapy. Tumor reliance on autophagy may increase in the presence of CD8^+^ T cell-mediated immune pressure, suggesting that autophagy inhibition could enhance anti-tumor immune responses. Yet clinical trials of autophagy inhibitors have yielded mixed outcomes, highlighting stage- and tumor-specific dependencies.

In neurodegenerative disorders, autophagy clears aggregation-prone proteins and damaged mitochondria and is broadly considered neuroprotective [222,223]. Enhancing autophagy via key regulators such as Beclin-1 and ATG proteins confers neuroprotection across multiple models, reinforcing its conserved role in neuronal maintenance [224,225,226]. Lysosomal dysfunction, genetic mutations (e.g., TDP-43, C9orf72), and age-related decline in autophagic capacity shift autophagy from protective to maladaptive. Excessive or dysregulated autophagy may contribute to neuronal self-digestion or increased stress signaling. Importantly, autophagy interacts tightly with ER stress and mitochondrial pathways. Impaired autophagy amplifies UPR signaling, which in chronic settings shifts from adaptive to pro-apoptotic outputs.

Comparative biology reveals that proteostasis networks are differentially tuned rather than uniquely altered in disease. Long-lived, cancer-resistant species like naked mole-rats and bowhead whales maintain sustained proteasome activity, efficient autophagic flux, and stable chaperone expression throughout life, buffering proteotoxic stress while preserving neuronal integrity [142,145]. These species exemplify evolutionary optimization of conserved proteostasis mechanisms, demonstrating that enhanced disease resistance arises from precise regulatory modulation rather than novel pathway acquisition.

In summary, proteostasis networks constitute a conserved stress-buffering system whose functional outcomes diverge sharply depending on cellular identity and regulatory context. In cancer, an increased proteostatic capacity supports survival amid persistent oncogenic stress while simultaneously creating vulnerabilities that can be targeted therapeutically. In neurons, insufficient, dysregulated, or chronically activated proteostasis contributes to aggregate accumulation, ER stress amplification, and inflammatory feedback loops. Therapeutic strategies must therefore navigate this duality, selectively attenuating proteostatic support in malignancies while restoring balance in NDDs.

### 4.4. Growth and Developmental Signaling Across Cellular Contexts

Evolutionarily conserved growth and developmental signaling pathways, including PI3K/Akt/mTOR, Wnt, Notch, Hippo, and TGF-β, coordinate essential processes such as cell proliferation, metabolism, differentiation, and tissue remodeling throughout metazoans. As illustrated previously for mTOR signaling (Section 2.1), these pathways are critical for development and regeneration [227,228,229,230,231,232]. However, when their activation becomes sustained, mistimed, or spatially misregulated, they generate divergent pathological outcomes in cancer and neurodegeneration.

These divergent effects reflect variations in cellular identity, regenerative capacity, and temporal regulation rather than differences in the pathways themselves.

#### 4.4.1. Wnt and Notch Signaling: From Cellular Plasticity to Pathological Persistence

Wnt and Notch pathways regulate cell fate determination, stemness, and tissue patterning across species [233]. In cancer, aberrant Wnt signaling supports sustained proliferation, invasion, and tumor stem cell maintenance, particularly in colorectal and brain cancers [234,235]. For example, in colorectal carcinoma, mutations in APC or CTNNB1 (β-catenin) drive constitutive pathway activation. Loss of APC function disrupts the β-catenin destruction complex, leading to nuclear accumulation of β-catenin and sustained transcription of proliferative gene programs. Similar hyperactivation is observed across several solid tumors. However, therapeutic targeting of Wnt has proven challenging. Because APC is a tumor suppressor, it cannot be directly restored pharmacologically. Consequently, attention has shifted toward downstream effectors such as TNIK, β-catenin–TCF interactions, and components of the destruction complex. Small-molecule inhibitors targeting these nodes have entered early clinical evaluation. Despite these advances, Wnt inhibition carries risks. The pathway is essential for normal stem cell maintenance in intestinal crypts and hematopoietic compartments, and systemic blockade frequently produces toxicity. Thus, the therapeutic window remains narrow.

In contrast, in neurodegenerative diseases, impaired or insufficient Wnt signaling has been implicated in synaptic dysfunction, reduced neurogenesis, and neuronal vulnerability. Experimental approaches therefore aim to enhance rather than inhibit Wnt activity to support neuronal survival. However, no Wnt-targeted therapies are currently approved in neurodegeneration, and the field remains largely preclinical. A critical unresolved question is whether observed Wnt alterations in aging brains represent pathogenic suppression or compensatory attempts at repair. This therapeutic asymmetry—Wnt inhibition in cancer versus Wnt enhancement in neurodegeneration—exemplifies the context-dependent polarity of conserved signaling pathways.

Notch signaling similarly illustrates duality. Notch signaling likewise enhances cancer stem cell programs, epithelial–mesenchymal transition (EMT), and therapy resistance [236,237]. These roles have motivated clinical development of γ-secretase inhibitors (GSIs), which block Notch receptor activation. However, broad Notch inhibition has shown modest efficacy and significant gastrointestinal and immune toxicities due to pathway suppression in normal tissues.

Complicating this landscape further, Notch can function as either an oncogene or a tumor suppressor depending on tissue context and mutational background. As a result, biomarker-driven stratification is increasingly recognized as essential for effective clinical deployment.

In the nervous system, Notch regulates adult neurogenesis, glial differentiation, and synaptic plasticity [238,239,240]. Dysregulation has been linked to impaired circuit stability and neuroinflammatory responses. Yet whether Notch activation in degenerative contexts is maladaptive or protective remains debated, and therapeutic modulation in neurodegenerative disease is still exploratory [241].

#### 4.4.2. Hippo Pathway: Regulating Size, Polarity, and Cell Fate Decisions

The Hippo pathway, first identified in *Drosophila*, is a conserved switch of organ size, cell proliferation, and apoptosis [242]. In cancer, loss of Hippo signaling results in nuclear accumulation of YAP/TAZ, activating oncogenic transcriptional programs that promote invasion and metastasis across diverse tumor types [243,244]. Additionally, disruption of epithelial polarity proteins like Dlg5 exacerbates Hippo pathway dysfunction, further driving malignancy via YAP-dependent mechanisms [245,246]. Notably, YAP/TAZ activity is also influenced by interactions with receptor tyrosine kinases and G-protein-coupled receptor-mediated growth pathways, integrating Hippo signaling into broader oncogenic networks that support proliferation and survival. Although pharmacologic inhibitors of YAP, including verteporfin, have shown preclinical promise, systemic targeting remains difficult due to the context-specific roles of YAP in normal tissues.

In parallel with its oncogenic roles, within the nervous system, Hippo signaling modulates neuronal survival, differentiation, and inflammatory processes [163,247]. Its dysregulation leads to abnormal apoptosis and glial activation, indicating that polarity-associated growth control mechanisms, conserved to maintain tissue integrity, can increase neuronal vulnerability to degeneration when improperly regulated [248]. Thus, growth-control mechanisms conserved to maintain tissue integrity can increase neuronal vulnerability when temporally misregulated or chronically activated.

#### 4.4.3. TGF-β Signaling: Mediating Homeostasis, Inflammation, and Disease Progression

TGF-β signaling is an evolutionarily conserved regulatory hub that integrates canonical Smad-dependent transcription with noncanonical pathways—including MAPK, PI3K/AKT, Rho GTPases, and NF-κB—to coordinate cell proliferation, differentiation, apoptosis, extracellular matrix remodeling, and immune modulation [249]. Its functional impact is profoundly stage- and context-dependent in both cancer and neurodegeneration.

Early in tumorigenesis, TGF-β suppresses proliferation and enforces growth restraint; however, in advanced cancers, it facilitates immune evasion, invasion, and metastatic remodeling through interactions with redox and inflammatory pathways [250,251,252]. This functional shift reflects changes in receptor signaling, Smad availability, and crosstalk with oncogenic networks. Interactions between TGF-β and redox pathways further amplify this complexity: TGF-β can modulate reactive oxygen species (ROS) production, while ROS can enhance TGF-β activation, forming feedback loops that either reinforce senescence and apoptosis or drive pro-metastatic remodeling depending on tumor stage and microenvironmental context. Such bidirectional signaling underscores why therapeutic inhibition of TGF-β has yielded mixed outcomes and remains limited by systemic toxicity and pleiotropic effects.

In the central nervous system, TGF-β supports synaptic stability and limits inflammation, exerting neuroprotective effects [253]. Canonical TGF-β/Smad signaling in astrocytes and microglia restrains excessive inflammatory activation and supports tissue repair. However, aging, chronic stress, or sustained inflammatory signaling can dysregulate this pathway. Reduced Smad responsiveness or aberrant TGF-β activation may impair clearance of protein aggregates, promote maladaptive gliosis, and contribute to synaptic dysfunction during neurodegeneration [254,255]. Thus, as in cancer, TGF-β signaling in the brain transitions from protective homeostatic regulation to maladaptive persistence when regulatory balance is lost.

The evolutionary diversification of Smad modules and noncanonical branches allows TGF-β signaling to adapt to diverse cellular demands. Yet this adaptability also increases vulnerability to context-specific dysregulation. In proliferative tissues, evasion of TGF-β growth suppression enables malignant expansion; in post-mitotic neural tissues, chronic or improperly resolved signaling can exacerbate inflammatory and fibrotic responses, impairing regeneration and accelerating degeneration.

Together, these growth and developmental signaling pathways illustrate a fundamental principle in disease biology: conserved pathways do not dictate disease specificity; rather, it is the regulatory context that shapes outcomes. Evolution has optimized these signaling networks to balance growth, plasticity, and tissue maintenance. Cancer exploits sustained pathway activation to drive expansion and survival, whereas neurodegeneration arises when these pathways are chronically engaged or improperly suppressed in post-mitotic cells.

Recognizing that evolution has tuned—not reinvented—these pathways provides a strategic framework for therapeutic precision, aiming to attenuate growth signals in cancer while restoring temporal and cell-type–specific signaling dynamics to preserve neuronal function.

### 4.5. Immune and Inflammatory Set Points as Disease Modulators

**Figure 5:**
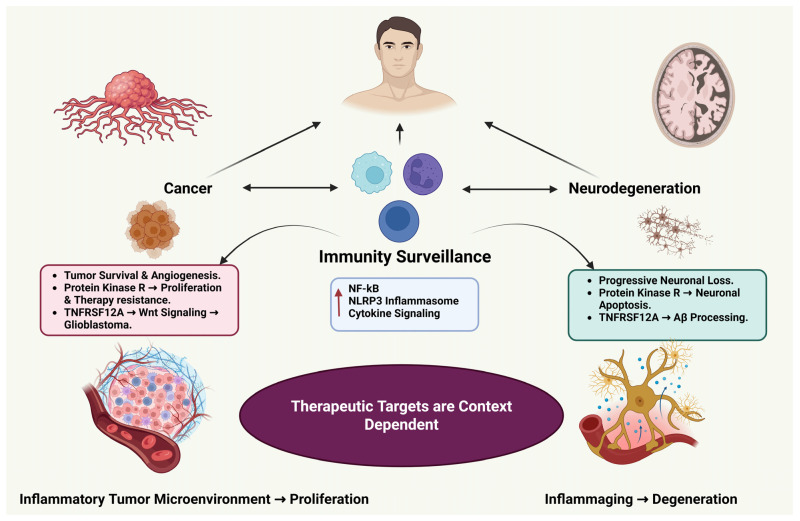
Schematic illustrating immune and inflammatory signaling as a central regulatory node balancing cancer progression and neurodegenerative processes.

Innate immune signaling establishes tissue-specific inflammatory thresholds shaping disease trajectories (Figure 5). NF-κB integrates cytokine, oxidative stress, and mitochondrial dysfunction signals to regulate immunity and survival [256]. Persistent NF-κB activation promotes angiogenesis, immune evasion, and metastasis in cancer [257,258], whereas sustained NF-κB signaling in microglia and astrocytes drives chronic neuroinflammation and synaptic failure in the CNS [259,260].

The NLRP3 inflammasome shows context-dependent roles in cancer [261] but is consistently pathogenic in neurodegeneration, activated by aggregated proteins and mitochondrial dysfunction, leading to chronic inflammation and pyroptotic cell death [262,263]. Cytokine networks further shape tumor-supportive microenvironments or drive synaptic dysfunction and neuronal loss [264,265,266].

Comparative and evolutionary analyses reveal that these immune mechanisms are deeply conserved rather than recent innovations. Immune checkpoint pathways, such as CD200–CD200R1 interactions, exemplify this conservation. Studies of transmissible cancers in Tasmanian devils show that immune evasion strategies exploiting conserved checkpoint signaling closely parallel those in human cancers, reflecting over 160 million years of evolutionary preservation in immune tolerance mechanisms that facilitate tumor survival across species [267]. This underscores that cancer progression often arises from exploitation of ancestral immune regulatory programs rather than novel pathway acquisition.

Similarly, excessive or misdirected innate immune activation is a conserved driver of neurodegeneration across taxa. In *Drosophila*, chronic overactivation of innate immune pathways—including NF-κB–dependent transcription and antimicrobial peptide expression in the brain—induces progressive neuronal loss, establishing a mechanistic link between immune hyperactivation and neurodegeneration conserved from invertebrates to mammals [268]. Mitochondrial dysfunction and oxidative stress function as conserved immune checkpoints, amplifying inflammasome activation and inflammatory signaling that heighten neuronal vulnerability across species [269].

Additional conserved immune regulators illustrate context-dependent divergence. Protein kinase R (PKR), an interferon-inducible kinase conserved among eukaryotes, modulates apoptosis, translational arrest, and NF-κB signaling. Dysregulated PKR activation drives neuronal apoptosis in neurodegeneration, while altered PKR activity in cancer influences proliferation, immune evasion, and therapy resistance [270]. Similarly, immune-associated tumor suppressors such as TNFRSF12A participate in shared regulatory networks affecting Wnt signaling and amyloid precursor protein processing, linking glioblastoma biology with AD progression through conserved immune–growth signaling crosstalk [271].

At an evolutionary level, the inverse epidemiological relationship between cancer and NDDs reflects trade-offs embedded within conserved immune, cell-cycle, and survival pathways. While proliferative tissues harness inflammatory signaling to promote expansion and repair, neurons—optimized for longevity and functional stability—accumulate damage under chronic immune activation [8]. Immune-associated protein quality control mechanisms, including chaperone-mediated autophagy, further modulate this balance by connecting inflammation, proteostasis, and neuronal survival within conserved pathways disrupted across neurodegenerative disorders [272,273,274,275,276].

Across genome maintenance, mitochondrial regulation, proteostasis, growth signaling, and immune control, a unifying principle emerges: conserved molecular pathways are not inherently oncogenic or neurotoxic. Their effects depend on cellular context, temporal dynamics, metabolic state, and proliferative capacity. Adaptive stress responses promote survival and expansion in dividing cells but culminate in degeneration in post-mitotic neurons. Therapeutic strategies must, therefore, target these pathways with cell-type specificity and temporal precision to avoid inadvertently exacerbating cancer or neurodegeneration (Supplementary Table S2 illustrates the context-dependent roles of conserved molecular pathways in cancer and neurodegeneration, emphasizing that disease-specific outcomes reflect differences in cellular context, proliferative capacity, metabolic demand, and temporal regulation rather than pathway novelty).

## 5. Systems-Level Integration of the Cancer–Neurodegeneration Paradox

The contrasting phenomena of uncontrolled cellular proliferation in cancer and premature cell loss in neurodegeneration reflect a systems-level dynamic rather than isolated molecular events. Systems biology offers a comprehensive framework to integrate multiscale data—from genes and proteins to cells, tissues, and species—to elucidate how conserved molecular networks are differentially regulated in these disease contexts. Advances in network biology, multi-omics integration, single-cell and spatial profiling, and comparative systems analysis have begun to clarify this paradox by revealing shared modules, regulatory hubs, and context-dependent network behaviors across cancer and neurodegenerative disorders.

### 5.1. Network-Based Integration of Cancer and Neurodegeneration

Network biology condenses complex omics data into interpretable disease modules representing coordinated molecular activities beyond single-gene effects [277]. Methods like co-expression networks, network propagation, and matrix factorization help identify both shared and unique modules across diseases. For example, Zhao et al. demonstrated that network integration can uncover conserved functional modules spanning multiple disease states by aggregating transcriptomic and proteomic datasets into coherent subnetworks [278]. Similarly, Greco et al. applied matrix factorization to lung cancer and Alzheimer’s disease transcriptomes, identifying cross-disease gene candidates—including ER, CDH1, and HDACs—that contribute to inverse comorbidity [279]. These shared modules were enriched in mitochondrial, immune, and metabolic pathways, indicating that divergence arises from regulatory reconfiguration of shared networks. Large-scale disease-gene network analyses by Zeighami et al. further support this, revealing distinct “tumor-related” and “neurodegenerative” co-expression clusters, with most disease-associated genes expressed within the same cell types across humans and mice, highlighting the deep conservation of cellular disease modules [280].

### 5.2. Multi-Omics and Cross-Disease Integration

Multi-omics integration strengthens mechanistic insight by linking genetic variation to downstream transcriptional, epigenetic, and metabolic states [278]. Cross-disease analyses combining Mendelian randomization, bulk and single-cell RNA sequencing, and functional validation have identified shared regulatory nodes between AD and glioblastoma, including TNFRSF12A, whose perturbation influences amyloid-processing and Wnt signaling pathways in tumor cells [271]. These findings illustrate how conserved growth and immune signaling modules are repurposed across divergent pathologies.

Cross-species multi-omic analyses by Foerster et al. further demonstrate that developmental and epigenetic programs can be reactivated in disease. Integration of transcriptomic and DNA methylation data from human glioblastoma and mouse neural stem cells identified regulators such as SFRP1 that modulate tumor epigenetic states and cellular quiescence [281]. Such analyses highlight how conserved regulatory frameworks are re-engaged in context-dependent ways to shape disease trajectories.

### 5.3. Single-Cell and Spatial Systems Biology

Single-cell and spatial omics technologies have revolutionized systems-level analysis by resolving cellular heterogeneity and microenvironmental context—critical determinants of disease outcomes. In cancer, single-cell RNA sequencing reveals clonal evolution, transcriptional plasticity, and immune heterogeneity within tumors, while spatial transcriptomics maps these states within tumor architecture [282,283,284]. In neurodegenerative diseases, single-cell profiling identifies vulnerable neuronal and glial subpopulations marked by mitochondrial dysfunction, inflammatory activation, or impaired proteostasis. Spatial omics further reveal how microglial and astrocytic states spatially correlate with amyloid plaques, tau pathology, or motor neuron degeneration in AD, PD, ALS, and FTD [285,286,287]. These studies demonstrate that conserved pathways—immune signaling, metabolic regulation, and stress responses—are deployed in spatially and temporally distinct patterns that shape divergent disease phenotypes.

### 5.4. Computational Modeling, Machine Learning, and Predictive Systems Biology

Computational modeling facilitates dynamic simulation of disease networks, capturing feedback loops and emergent behaviors not evident from static data [288]. Bayesian and graphical network models have identified master regulators such as TYROBP in microglial activation in AD [289], while simulations of NF-κB and mTOR pathways reveal self-sustaining inflammatory and autophagy-inhibitory loops accelerating neurodegeneration [290]. Machine learning approaches integrate molecular, imaging, and clinical data to predict disease trajectories and stratify patients. AI models combining genetic, proteomic, and MRI data accurately forecast AD progression, while similar frameworks in cancer identify therapy-resistant states and optimize treatment combinations [291,292]. Genome-scale metabolic modeling further reveals conserved metabolic dependencies influencing immune and stress pathways across both diseases [293,294].

### 5.5. Cross-Species Comparative Systems Biology

Comparative systems biology exploits evolutionary conservation to identify resilience and vulnerability pathways across species. Model organisms—from *Drosophila* and *C. elegans* to rodents and non-human primates—have been pivotal in dissecting shared mechanisms including DNA damage response, proteostasis, RNA metabolism, and immune signaling [295,296,297]. Cross-species analyses reveal that genes regulating proliferation in cancer often have orthologs controlling neuronal survival or death, explaining epidemiological inverse comorbidity patterns [11]. At a systems level, these conserved mitochondrial, oxidative stress, and inflammatory modules emerge across taxa, with alterations in mtDNA copy number and immune activation serving as shared disease markers [298,299]. Such cross-species convergence provides a strategy to prioritize therapeutic targets that are evolutionarily constrained yet differentially regulated across tissues.

### 5.6. Conceptual Synthesis: Resolving the Paradox

The cancer–neurodegeneration paradox arises from shared molecular networks governing cell survival, stress adaptation, and quality control, which are differentially regulated depending on cellular context. Cancer exploits sustained activation of these networks to promote proliferation and evade cell death, whereas neurons—optimized for longevity and functional stability—are vulnerable to damage when these pathways are chronically engaged. Systems-level integration across omics layers, cell types, and species reveals that disease specificity depends not on pathway novelty but on network state, temporal dynamics, and cellular constraints. These insights lay the groundwork for identifying dual-purpose biomarkers and developing therapies that selectively modulate conserved pathways—suppressing maladaptive survival in cancer while enhancing resilience in neurodegeneration.

## 6. Bioinformatics and Cross-Species Genomic Approaches in Cancer and Neurodegeneration

Bioinformatics and cross-species genomic methodologies have become essential for elucidating the shared and distinct molecular mechanisms driving cancer and neurodegeneration. By harnessing evolutionary conservation alongside large-scale genomic and multi-omics data integration, these approaches transcend single-gene studies to enable systems-level insights. Comparative oncology and neurogenomics reveal that core regulatory networks—governing genome stability, cell cycle control, proteostasis, metabolism, and immune signaling—are deeply conserved but differentially repurposed according to tissue identity and disease state. Cross-species alignment therefore enables the identification of evolutionarily constrained nodes that may serve as robust translational targets or biomarkers.

### 6.1. Genome-Level Comparative Oncology and Evolutionary Constraint

Comparative oncology employs genome-scale bioinformatics to explore cancer susceptibility and resistance across species by analyzing conserved and divergent genomic regions linked to disease risk. Ortholog identification and sequence alignment tools, such as Ensembl Compara, facilitate gene tree construction and pairwise or multiple genome alignments, enabling cross-species gene comparisons [300].

A major advance in this field is the Zoonomia Project, which assembled high-quality genomes for 131 mammals and generated whole-genome alignments across 240 species, covering over 80% of mammalian families [301]. This extensive dataset serves as a foundation for conservation-based analyses of disease-associated genes. Phylogenetic profiling further predicts cancer susceptibility by assessing evolutionary constraint. For example, Nair et al. applied BLAST-derived conservation scores across 193 vertebrates, revealing that genes highly conserved in long-lived, cancer-resistant species including elephants, whales, and bats are enriched for DNA repair, cell-cycle regulation, and immune pathways [302]. Notably, these genes significantly overlap with established human cancer driver genes. This phylogeny-informed approach supports a genomics-based predictor of intrinsic cancer resistance grounded in purifying selection rather than species-specific innovations.

Cancer, an ancient disease with deep evolutionary roots, exhibits variable incidence across taxa, with some species evolving remarkable resistance mechanisms through natural selection [303]. Protein evolution in cancer is shaped by the roles of these proteins in protein interaction networks: proteins at the center of highly connected groups are conserved, while those linking different groups are more adaptable. This suggests that cancer-related changes often happen by altering connections between protein groups, similar to domain shuffling in protein evolution [304].

Comparative oncology also addresses Peto’s paradox, which notes that large, longer-lived animals have evolved better cancer defenses. For example, elephants have extra copies of the tumor suppressor gene TP53 and enhanced immune regulation, helping them resist cancer [305,306].

Beyond tumor suppressors, evolutionary adaptation extends to immune defense genes. Comparative genomics reveals that highly conserved gene structures enable cross-species identification of novel resistance genes protecting against both malignancies and infectious diseases [307]. Genetic variations such as aneuploidy also influence cancer progression and therapeutic vulnerabilities, modulated by species-specific genomic contexts and environmental factors [308].

Viewing cancer as an evolutionary and speciation-like process emphasizes the genomic plasticity and clonal evolution of cancer cells within hosts, framing cancer biology within broader evolutionary dynamics. This perspective opens therapeutic avenues targeting mechanisms rooted in cancer’s evolutionary origins and genomic complexity [309]. Furthermore, recognizing cancer as a multispecies process involving host and microbial genomes expands comparative oncology’s scope, implicating host–microbe interactions in cancer evolution and treatment strategies [310].

In summary, genome-level comparative oncology integrates evolutionary principles and cross-species genomic analyses to uncover natural cancer resistance mechanisms and evolutionary constraints shaping susceptibility. Insights from diverse organisms promise to advance cancer prevention, diagnosis, and therapy by revealing fundamental biological rules and innovative adaptations refined over millions of years [303,304,305].

### 6.2. Canine Cancer Models as Translational Genomic Platforms

#### 6.2.1. Comparative Genomics of Canine Tumors

Domestic dogs serve as valuable translational models due to their high incidence of spontaneous cancers, shared environmental exposures with humans, and intact immune systems. Rodrigues et al. performed large-scale cancer genome sequencing on 671 dogs spanning 96 breeds and 23 tumor types, revealing mutational hotspots conserved with human cancers [311]. Recurrent oncogenic alterations were detected in canonical drivers such as PIK3CA, KRAS, NRAS, BRAF, KIT, and EGFR. Tumor-specific mutations paralleled those in human malignancies, including NRAS G61R and PIK3CA H1047R in hemangiosarcoma, ERBB2 V659E in pulmonary carcinoma, and BRAF V588E (homologous to human V600E) in urothelial carcinoma [311].

Similarly, Shao et al. identified DLG2 as a tumor suppressor gene through comparative genomic analysis of spontaneous osteosarcoma in humans and dogs, with copy number loss observed in 42% of human and 56% of canine tumors, implicating conserved polarity and synaptic scaffold genes in oncogenesis [312].

#### 6.2.2. Transcriptomic and Subtype-Level Conservation

Whole-exome and transcriptomic profiling of 191 canine mammary tumors by Kim et al. demonstrated strong molecular resemblance to human breast cancer. PIK3CA mutations occurred frequently (43.1%), with PI3K–Akt pathway alterations present in 61.7% of cases. Transcriptomic clustering revealed three canine tumor subtypes, including one closely resembling basal-like human breast cancer, characterized by EMT activation, low claudin expression, and poor prognosis [313].

#### 6.2.3. Canine Genomic Resources and Public Repositories

Dogs exhibit cancer incidence approximately five times higher than humans [314], with over one million cases treated annually in the US [315], facilitating longitudinal biomarker studies through repeated sampling of blood, urine, and tissues.

The CanFam4 reference genome [316] markedly enhanced canine genomic resolution, particularly in immunogenomic regions such as the major histocompatibility complex (DLA), T-cell receptor loci, and hundreds of cancer-related genes cataloged in COSMIC. Concurrently, the Dog10K Consortium sequenced ~2000 canids across 321 breeds [317], establishing the most comprehensive canine variant reference to date.

Public repositories like the Integrated Canine Data Commons (ICDC) aggregate harmonized genomic and clinical datasets of spontaneous canine cancers [318]. The ICDC-Glioma collection integrates whole-genome sequencing, whole-exome sequencing, RNA-seq, DNA methylation, MRI, and histopathology data, enabling direct comparative analyses between canine and human gliomas. The Cancer Imaging Archive (TCIA) further supports cross-species imaging-genomics integration.

Comparative studies consistently show conservation of chromosomal aberrations, copy number alterations, and transcriptional subtypes between canine and human tumors. For example, recurrent DNA copy number changes in canine osteosarcoma involve orthologous regions containing RUNX2 and PTEN [319]. Canine mammary tumors also mirror human molecular subtypes, including luminal and basal-like signatures defined by transcriptomic and genomic profiling [313,320,321]. Intracranial tumors and mucosal melanoma display conserved syntenic alterations and shared candidate oncogenes, reinforcing their translational relevance [322,323].

Advances such as high-resolution canine CGH arrays enable precise mapping of cytogenetic abnormalities to genomic coordinates, enhancing cross-species tumor cytogenetics [324]. Canine cancer research benefits from publicly available genomic datasets and bioinformatics platforms that support multi-omics integration and analysis, paralleling human resources like The Cancer Genome Atlas. These databases facilitate comparative oncogenomics, subtype characterization, and identification of conserved oncogenic pathways, promoting translational discoveries in diagnostics and therapeutics [325].

In summary, comparative genomic and transcriptomic analyses of canine tumors reveal significant conservation of molecular subtypes, gene expression signatures, and oncogenic pathways with human cancers. The canine model, supported by comprehensive genomic resources and public repositories, advances understanding of cancer biology and facilitates precision oncology development for both species [326,327].

### 6.3. Bovine Models: Environmental Carcinogenesis and Leukemia Networks

#### 6.3.1. Bovine Urothelial Carcinoma as an Environmental Cancer Model

Cattle grazing on bracken fern (BF) develop spontaneous urothelial carcinoma driven by the carcinogen ptaquiloside (PT). Wong et al. demonstrated that BF-exposed cattle exhibit exceptionally high mutational burdens with distinctive mutational signatures characterized by T/A substitutions and deletions. In vitro assays confirmed PT as the causative mutagen. These findings have direct implications for human bladder, esophageal, and gastric cancers linked to BF exposure through contaminated food and water sources.

#### 6.3.2. Bovine Leukemia Virus and Network-Level Oncogenesis

Bovine leukemia virus (BLV), a retrovirus closely related to human T-cell leukemia virus type 1 (HTLV-1), serves as a valuable model for studying viral oncogenesis and leukemia networks [328]. Although natural BLV infection in cattle results in low tumor incidence, experimental infection in sheep induces overt leukemia, facilitating investigation of leukemogenesis mechanisms. Klener et al. employed cross-species ovine-to-human microarrays to analyze BLV Tax-responsive transcriptional changes, revealing deregulation of apoptosis, DNA repair, transcriptional regulation, proto-oncogenes, and Rho GTPase signaling [329]. This broad perturbation of interconnected oncogenic pathways, rather than lineage-specific effects, mirrors molecular events in human B-cell malignancies, underscoring the translational relevance of bovine models.

Environmental carcinogens also contribute to leukemogenesis in bovine systems. Benzene, a recognized human carcinogen, has been studied for its impact on hematopoietic signaling pathways. Animal models indicate that in utero benzene exposure elevates tumor incidence in offspring, implicating disruption of pathways such as Hedgehog, Notch/Delta, Wingless/Integrated, and NF-κB in leukemia development. However, mechanistic understanding remains incomplete, warranting further research [330].

Bovine papillomaviruses (BPVs) further exemplify interactions between viral oncogenesis and environmental factors. BPVs induce benign hyperplastic lesions that may progress to malignancy, especially when combined with environmental co-factors like immunosuppressants and carcinogens from bracken fern. This synergy between viral oncoproteins and environmental carcinogens parallels mechanisms observed in human papillomavirus-associated cancers. Detailed studies of BPV molecular biology and oncoprotein functions provide insights into environmentally influenced tumorigenesis in bovine models [331].

More broadly, bovine and other animal models have significantly advanced understanding of chemical and environmental carcinogenesis. These models have elucidated multistage tumor development, identified key genetic and epigenetic alterations driving cancer initiation and progression, and explored genotype–environment interactions, inflammation, and prevention strategies. Such insights are directly applicable to human environmental carcinogenesis [332,333,334].

In summary, bovine leukemia and papillomavirus models illustrate how viral oncogenes, environmental carcinogens, and host signaling networks converge to drive malignancy. By recapitulating conserved pathways—apoptosis, DNA repair, inflammatory signaling, and developmental cascades—these systems provide translational insight into virus-associated and environmentally influenced cancers in humans [329,330].

### 6.4. Murine Cross-Species Genomics and Multi-Omics Integration

Murine models remain foundational in cancer bioinformatics due to their genetic tractability and extensive dataset availability. Comparative genomic microarray analyses (CGMA), combined with pathway enrichment tools such as KEGG and Reactome, have demonstrated that tumors in mice, rats, and humans share dysregulated processes including apoptosis, cell cycle control, differentiation, and metabolism [335].

Cross-species integration studies highlight both conservation and limitations. Connolly et al. compared PDGF-driven gliomas across rats, mice, dogs, and humans, identifying a conserved neoplastic core centered on signal transduction and invasion pathways [336]. However, immunosuppressive M2 macrophage signatures were detected in rat and human gliomas but not in mouse models, underscoring species-specific differences in tumor microenvironment recapitulation. Integrative alignment frameworks such as AJIVE, Celligner, PRECISE/TRANSACT, and MOFA further enhance translational fidelity by disentangling shared biological signals from cohort-specific noise across mouse models, cell lines, and human tumors [337].

Cross-species oncogenomic studies have illuminated conserved genetic and molecular features relevant to cancer progression and therapeutic stratification. For instance, analyses of chromosomally unstable murine tumors revealed genomic alterations closely mirroring those in human cancers, including T-cell acute lymphoblastic leukemia/lymphoma (T-ALL). Shared deletions in FBXW7 and PTEN, alongside recurrent syntenic amplifications and deletions across human hematopoietic, mesenchymal, and epithelial tumors, highlight biological processes driven by orthologous genetic events, validating the use of genomically unstable murine models for human cancer gene discovery [338].

Carcinogen-induced oral squamous cell carcinoma (OSCC) models demonstrate conserved alterations in Trp53, MAPK, PI3K, NOTCH, and JAK/STAT pathways, and identify metastasis-associated transcriptional signatures predictive of aggressive human disease [339].

Cross-species analyses also uncover conserved tumor suppressors and subtype parallels. For instance, integrated human–canine–murine osteosarcoma studies identified DLG2 as a novel tumor suppressor. Following its identification through comparative genomics, functional studies in DLG2-deficient cell lines and osteoblast-specific knockout mouse models demonstrated accelerated tumor development, impaired cell cycle regulation, and enhanced migratory capacity. These findings demonstrate that polarity-associated scaffold proteins operate within evolutionarily conserved tumor suppressor networks, reinforcing the translational value of cross-species validation [312].

In breast cancer, transcriptomic classification of murine models revealed subtypes closely aligned with human basal-like and HER2-enriched tumors, supporting subtype-specific translational modeling [340]. Additionally, comparative genomics has revealed age-specific gene expression signatures and progression markers in breast cancer among young women, involving cell cycle and invasion-related genes, validated by multiple approaches [341]. Likewise, integrative genomic comparisons in hepatocellular carcinoma demonstrated that the STAM murine model recapitulates aggressive human HCC molecular features, whereas other models diverge, emphasizing the importance of careful model selection [342].

Collectively, these examples demonstrate how murine cross-species genomics and multi-omics integration identify conserved genetic alterations, molecular pathways, and transcriptional programs that enhance understanding of tumor biology and improve preclinical modeling, ultimately facilitating translational advances in cancer therapy.

### 6.5. Proteomic, Phosphoproteomic, and Epigenomic Cross-Species Analysis

Proteomic and phosphoproteomic profiling extends functional understanding beyond genomic data by elucidating dynamic signaling networks. Brubaker et al. integrated murine phosphoproteomics with human tumor genomics, revealing tissue- and mutation-specific rewiring of KRAS signaling, including differential activation of MAPK, TGF-β, angiogenesis, and EMT pathways [343].

Epigenomic cross-species alignment represents a growing frontier. Foerster et al. (2025) employed pseudotime alignment (ptalign) to map human glioblastoma single-cell RNA-seq profiles onto mouse neural stem cell activation trajectories, uncovering conserved dysregulation of SFRP1 at the quiescence–activation transition [281]. Restoration of SFRP1 reprogrammed the tumor DNA methylome and extended survival in murine models, highlighting conserved epigenetic control of tumor growth [281].

Large-scale proteomic and phosphoproteomic studies have characterized molecular heterogeneity in cancers. For example, profiling adenocarcinoma of the esophagogastric junction identified distinct proteomic subtypes with altered kinase-phosphosubstrate networks, revealing hundreds of potential druggable targets to inform precision therapies [344]. Similarly, analyses of Clinical Proteomic Tumor Analysis Consortium (CPTAC) datasets across multiple cancers identified phosphorylation signatures and recurrent activation of kinases such as Aurora kinase A (AURKA) and NEK2, common to breast, colon, lung, ovarian, and endometrial tumors [345]. Integrated platforms like the ProteoCancer Analysis Suite (PCAS) leverage these datasets to pinpoint oncogenic mechanisms and therapeutic candidates with cross-cancer translational relevance [346].

Cross-species phosphoproteomic and epigenomic analyses also illuminate the intersection of aging and cancer. Tools such as PTMoreR map conserved phosphorylation events across mammals, identifying phospho-signaling modules associated with aging phenotypes [347]. Single-cell epigenomic studies reveal overlapping transcriptomic and methylation signatures between aging tissues and cancer, suggesting that epigenetic reprogramming alters cellular identity and increases disease susceptibility [347,348].

Epigenomic regulation also critically influences cancer immunometabolism by modulating metabolic states of tumor and immune cells, thereby affecting tumor progression and immune evasion. Targeting epigenetic modifications presents a promising approach to reprogram tumor immunometabolism and enhance immunotherapy efficacy [349]. At the organismal level, cellular senescence exemplifies this duality: while initially tumor suppressive, senescent cells contribute to chronic inflammation and tissue dysfunction, linking aging, immune remodeling, and cancer risk [24].

Collectively, cross-species proteomic, phosphoproteomic, and epigenomic analyses have illuminated conserved molecular pathways underlying aging and cancer, identifying actionable therapeutic targets and biomarkers for disease prevention and treatment across species.

### 6.6. Bioinformatics Approaches for Cross-Species Neurodegeneration Analysis

Cross-species bioinformatics in neurodegeneration integrates multi-omics datasets—including genomics, transcriptomics, proteomics, metabolomics, and microbiome profiles—to elucidate conserved disease mechanisms. Comparative transcriptomic studies of mouse models of AD and human patients reveal closer resemblance to late-onset AD, sharing immune and lysosomal regulators such as SLC11A1, CD14, CD33, and C1QB, alongside conserved transcription factor networks [350,351].

Network alignment techniques have uncovered conserved protein–protein interaction subnetworks in AD [352] across humans and *C. elegans*. Complementary bioinformatics tools applied to redox proteomics, mass spectrometry, and metabolomics further clarify pathways involving oxidative stress, mitochondrial dysfunction, and neuroinflammation.

Canine neurodegeneration models, which naturally develop amyloid and tau pathologies, facilitate integrative analyses combining brain omics with gut microbiome and metabolomic data, advancing understanding of conserved gut–brain axis mechanisms [352]. In murine neurodegeneration research, integration of multi-omics data with imaging and machine learning enables disease state classification, biomarker identification, and progression prediction, while also revealing translational limitations inherent to these models [353].

Comparative biomarker analysis is a key bioinformatics strategy, integrating molecular profiles such as proteins, RNAs, and metabolites associated with neurodegeneration across species. Blood-based biomarkers including amyloid-β peptides and phosphorylated tau, extensively studied in human AD, are evaluated in animal models to confirm their conservation and pathological relevance [354,355]. Similarly, markers like neurofilament light chain provide comparative measures of neuronal damage.

Extracellular vesicle (EV) and exosome analyses represent another important approach. Bioinformatics characterizes EV cargo across neurodegenerative models, revealing conserved roles in disease progression and biomarker potential. These studies demonstrate EV-mediated propagation of pathological proteins and microRNAs, including inflammatory cytokines, conserved across species [356,357].

MicroRNA profiling further enhances cross-species neurodegeneration research by identifying conserved regulatory patterns implicated in neurogenesis and neuronal loss. Advanced computational tools facilitate integration across species, uncovering candidate biomarkers and therapeutic targets despite the evolving nature of this field [358].

Lipidomic bioinformatics focuses on sphingolipid metabolism alterations linked to neuronal survival and death. Cross-species analyses reveal conserved sphingolipid profile shifts in neurodegenerative models, connecting lipid dysregulation with oxidative stress and neurodegeneration pathways [359].

Bioinformatics also aids in dissecting molecular pathways affected by neurotoxic exposures with cross-species relevance, such as manganese-induced neurodegeneration. Integrative omics approaches identify therapeutic targets and elucidate toxicity mechanisms critical for translational applications from model organisms to humans [360].

Cross-species neurodegeneration analyses encompassing murine, bovine, canine, parrot, and whale models highlight both conserved biological processes and lineage-specific gene expression profiles through transcriptomic and signaling pathway studies. For instance, single-cell transcriptomics of SOD1 ALS patient-derived motor neurons in mice identified TGF-β pathway activation, specifically SMAD2, as a key driver of neurodegeneration, demonstrating conserved dysregulated pathways across ALS variants [361].

Canine transcriptomic studies reveal immune signaling upregulation, such as JAK-STAT and MAPK pathways, paralleling human inflammatory and neurodegenerative conditions, confirming conservation of immune-mediated disease progression [362]. Marine mammal transcriptomics provide insights into longevity and neurodegeneration resistance. Gray whale brain transcriptomes show elevated expression of genes associated with DNA repair, ubiquitination, apoptosis, and immune responses, consistent with neuroprotective adaptations [363]. Similarly, bowhead whales exhibit altered expression in insulin signaling and lipid metabolism pathways, linking metabolic adaptations to neurodegenerative resilience and extended lifespan [142]. Though less explored in parrots and bovines for neurodegeneration, cross-species transcriptomic analyses highlight species differences in pathways like vascular senescence across rats, monkeys, and humans, emphasizing the importance of selecting appropriate models based on pathway conservation.

Collectively, these cross-species bioinformatics studies reveal that while humans may exhibit unique gene expression profiles in adult hippocampal neurogenesis, fundamental processes such as DNA repair, immune activation, and oxidative stress responses are conserved. Leveraging these insights improves model selection and guides intervention development across species, enriching neurodegenerative disease research from murine models to whales.

### 6.7. Bioinformatics Tools and Resources for Cross-Species Analysis

A comprehensive suite of bioinformatics tools and resources underpins cross-species research in cancer and neurodegeneration, facilitating the integration and comparative analysis of multi-omics data across diverse species and disease contexts (Table 1). Genomic and orthology databases such as Ensembl Compara, NCBI Gene, and Mouse Genome Informatics enable precise mapping and comparison of orthologous genes, while expression alignment and integration tools—including AJIVE, JIVE, Celligner, PRECISE/TRANSACT, and MOFA—allow harmonization of transcriptomic and proteomic datasets by distinguishing shared biological signals from cohort-specific noise [337,364].

Network and pathway analysis platforms like Cytoscape, STRING, network alignment algorithms, Gene set enrichment analysis (GSEA), Enrichr, and ReactomePA support the identification of conserved molecular interactions and enriched biological pathways, enhancing the interpretation of complex cross-species datasets. Proteomic and epigenomic analyses benefit from specialized tools such as KiPNA for kinase activity profiling, Bismark and MethyKit for DNA methylation analysis, and LiftOver for coordinate conversion between genomes. Public data repositories—including TCGA, CPTAC, CCLE, GEO, SRA, ProteomeXchange, and ICDC—provide extensive multi-omics datasets critical for comparative studies.

In cancer research, resources like The Cancer Genome Atlas (TCGA) offer large-scale multi-omics data encompassing somatic mutations, gene and protein expression, and epigenetic modifications across thousands of tumors, facilitating cross-species comparisons of tumorigenic processes [325,365]. Tools such as ReactomeGSA enable automated mapping of species-specific data onto a unified pathway framework, allowing integrated analysis of transcriptomic and proteomic datasets across species to elucidate conserved biological mechanisms, including immune cell dynamics within tumor microenvironments [366,367]. Cloud-based platforms like Galaxy and DNAnexus streamline multi-omics data processing and integration, supporting precision oncology research enhanced by AI and machine learning approaches for biomarker discovery and therapeutic target identification [367].

For NDDs, bioinformatics efforts focus on mining and integrating complex omics datasets such as epigenomics, transcriptomics, proteomics, metabolomics, lipidomics, and spatial omics—to uncover molecular signatures and pathways implicated in disorders like AD and PD [368]. Cross-species systems bioinformatics approaches leverage data from animal models alongside human datasets to model disease mechanisms and identify candidate biomarkers, facilitating translational insights [369]. The analysis of microRNAs has become particularly important, with dedicated tools enabling their functional characterization and validation across species, highlighting their roles as regulators and biomarkers in neurodegeneration [358]. Integrative bioinformatics frameworks support the synthesis of heterogeneous data, aiding the identification of early causal events and mechanistic pathways that are otherwise difficult to resolve, thereby advancing understanding of neurodegenerative disease biology [369].

Bioinformatics and cross-species genomic approaches collectively demonstrate that cancer and neurodegeneration originate from conserved molecular frameworks shaped by distinct evolutionary, cellular, and environmental pressures. Comparative genomics uncovers fundamental disease modules, while multi-omics integration elucidates complex regulatory hierarchies. Cross-species alignments differentiate universally conserved mechanisms from species-specific modifiers. This comprehensive framework enhances translational insights, enabling targeted biomarker identification and the development of therapeutic strategies that precisely modulate shared pathways to inhibit cancer progression while safeguarding neuronal function.

## 7. Translational Insights and Future Directions

**Figure 6:**
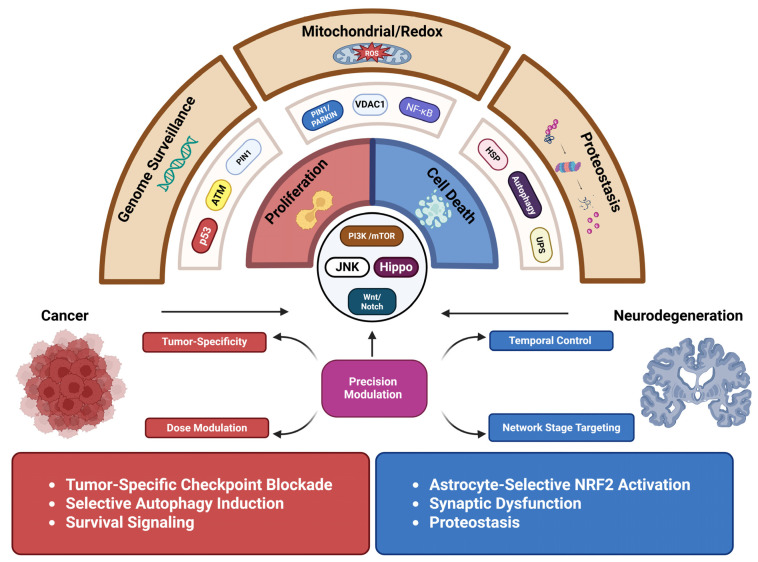
Precision Modulation of Conserved Pathways Across Cancer and Neurodegeneration.

The divergent cellular outcomes of unchecked growth in cancer versus neuronal loss in neurodegeneration reflect a systems-level imbalance rather than isolated molecular defects. The cancer–neurodegeneration paradox underscores how deeply conserved molecular networks—regulating cell survival, stress adaptation, metabolism, immune signaling, and proteostasis—are differentially engaged depending on cellular identity, tissue microenvironment, and life stage. This complexity challenges reductionist therapeutic approaches and necessitates strategies that acknowledge evolutionary trade-offs inherent within shared biological systems (Figure 6).

A key insight is that conserved pathways are not inherently oncogenic or neurotoxic; rather, their pathological effects emerge from context-dependent dysregulation. For example, p53 activation prevents cancer in dividing cells but induces apoptosis in neurons. Similarly, mTOR inhibition enhances autophagy in neurodegeneration but may impair immune competence and metabolism if applied systemically. Therefore, interventions require nuanced calibration rather than simple on/off modulation.

Among conserved pathways, autophagy and proteostasis regulators are promising targets in neurodegeneration, where partial restoration of degradative capacity can alleviate proteotoxic stress without widespread pathway activation. NRF2 modulation, especially via astrocyte-selective activation, may boost neuronal redox balance while minimizing oncogenic risk. Context-specific mTOR modulation, such as intermittent or low-dose regimens, holds potential to enhance neuronal resilience without compromising systemic functions. Selective immune recalibration, avoiding broad suppression or activation, could balance tumor surveillance with neuroprotection. Conversely, global NRF2 activation, systemic mTOR inhibition, broad immune checkpoint blockade, and pan-Wnt or Notch pathway suppression pose significant risks due to their essential roles in tissue regeneration, immune defense, and stem cell maintenance, limiting therapeutic windows.

Previous therapeutic failures largely stem from insufficient specificity. mTOR inhibitors caused metabolic side effects by affecting both tumor and normal tissues; HSP90 inhibitors triggered compensatory heat shock responses reflecting network adaptability; broad anti-inflammatory treatments failed due to immune activation’s dual protective and pathological roles; and γ-secretase inhibitors caused toxicity through systemic Notch suppression. These outcomes highlight that the conserved pathways function within adaptive networks resistant to uniform inhibition, emphasizing the need to target disease-specific network states rather than pathways alone.

Feasible precision strategies include cell-type–specific delivery methods (e.g., viral vectors, nanoparticles, antibody-drug conjugates), temporal modulation through intermittent dosing or stage-specific interventions, circuit-level targeting informed by single-cell and spatial transcriptomics, genotype-informed patient stratification focusing on mutations like KEAP1 or TP53, and combination therapies preserving stress resilience in healthy tissues. Emerging gene-editing and RNA-based therapeutics enable refined, compartmentalized pathway modulation.

Advances in comparative genomics and multi-omics studies demonstrate that longevity and cancer resistance arise from finely tuned regulatory balance rather than pathway elimination. Long-lived species maintain balanced p53 activity, enhanced proteostasis, and mitochondrial resilience without inducing neurodegeneration, suggesting that restoring regulatory equilibrium is key to disease prevention.

Future translational approaches should adopt a network-state medicine framework, integrating multi-omics and artificial intelligence to define cell-type–specific vulnerability signatures and identify master regulators of disease-specific network configurations. Digital twin modeling can facilitate in silico testing of pathway modulation strategies prior to clinical application, reducing risk and improving precision.

Ultimately, addressing the cancer–neurodegeneration paradox requires tissue-specific, age-aware, stage-dependent, and dosage-precise interventions that modulate conserved pathways to restore physiological balance without destabilizing interconnected systems. The central translational question shifts from whether to inhibit or activate a pathway to how, where, and when to tune it for optimal therapeutic outcomes.

## 8. Conclusions

Cancer and neurodegeneration, traditionally seen as opposing disease processes, originate from the dysregulation of shared, evolutionarily conserved molecular networks. Their distinct manifestations are shaped not by the pathways themselves but by the cellular environment, temporal regulation, and organismal constraints. This review is focused on the evolutionarily conserved pathways across species that control genome stability, metabolism, proteostasis, and immune regulation in proliferative or post-mitotic cells. Consequently, the cancer–neurodegeneration paradox reflects a core biological trade-off between regeneration and degeneration, with antagonistic pleiotropy serving as an integrated evolutionary mechanism influencing disease susceptibility.

Embracing this integrative perspective transforms the cancer–neurodegeneration paradox into a strategic opportunity for translational advances. By aligning therapeutic development with evolutionary principles and systems biology, future interventions may extend human healthspan by concurrently restraining malignant growth and preserving neuronal resilience. If cancer and neurodegeneration represent divergent outcomes of the same conserved molecular networks, can emerging therapies be designed to retune—rather than silence—these pathways, achieving a balance between effective tumor suppression and sustained neural preservation?

## Figures and Tables

**Table 1:** A summary of bioinformatics tools and resources for cross-species analysis in cancer and neurodegeneration as discussed in Section 6.7.

Category	Tool/Resource	Primary Function	Application in Cross-Species Analysis
Orthology & Genomics Databases	Orthology & Genomics Databases	Ensembl Compara	Cross-species gene comparison
	NCBI Gene	Gene annotation	Comparative genomics
	Mouse Genome Informatics	Mouse-human gene mapping	Translational model validation
Multi-Omics Integration Tools	AJIVE	Joint variation analysis	Align transcriptomic datasets
	Celligner	Tumor–cell line alignment	Model–human matching
	PRECISE/TRANSACT	Cross-cohort harmonization	Noise reduction across species
	MOFA	Multi-omics factor analysis	Integrative disease module detection
Network & Pathway analysis	Cytoscape	Network visualization	Conserved interaction mapping
	STRING	PPI Networks	Cross-species module conservation
	GSEA/Enrichr	Pathway enrichment	Functional module identification
	ReactomePA	Pathway mapping	Species normalized pathway analysis
Proteomic/Epigenomic tools	KiPNA	Kinase activity inference	Phospho network comparison
	Bismark	DNA methylation mapping	Epigenomic conservation
	MethyKit	Differential methylation	Aging and cancer epigenetics
	LiftOver	Genome coordinate conversion	Cross-genome annotation
Public Multi-Omics Repositories	TCGA	Human tumor multi-omics	Cross species cancer benchmarking
	CPTAC	Tumor proteomics	Cross cancer phosphoproteo-mic-profiles
	CCLE	Cancer cell lines	Model comparison
	GEO/SRA	Transcriptomic datasets	Cross-condition reanalysis
	ICDC	Canine cancer genomics	Comparative oncology
Cloud & Workflow Platforms	Galaxy	Reproducible pipelines	Multi-omics integration
	DNAnexus	Scalable genomic processing	AI-enabled precision oncology
Neurodegeneration-Specific Tools	miRNA analysis platforms	Regulatory RNA mapping	Cross-species biomarker discovery
	Spatial omics integration frameworks	Tissue-context mapping	Neuroimmune spatial profiling

## Data Availability

No new data were created or analyzed in this study. Data sharing is not applicable to this article.

## Supplementary Materials

The following supporting information can be downloaded at: https://www.mdpi.com/article/10.3390/ijms27072989/s1.

## Abbreviations

The following abbreviations are used in this manuscript:

AD	Alzheimer’s Disease
PD	Parkinson’s Disease
ALS	Amyotrophic Lateral Sclerosis
HD	Huntington’s disease
NDDs	Neurodegenerative diseases
PM	Post mitotic
AR	Androgen Receptor
CSC	Cancer Stem cell
NK	Natural Killer cell
EMT	Epithelial–mesenchymal transition
UPS	Ubiquitin–proteasome system
UPR	Unfolded protein response
ICDC	Integrated Canine Data Commons
EV	Extracellular Vesicle
TCGA	The Cancer Genome Atlas
BLV	Bovine leukemia virus
CGH	Comparative Genomic Hybridization
CGMA	Comparative Genomic Microarray Analysis
